# Cholesterol metabolism: molecular mechanisms, biological functions, diseases, and therapeutic targets

**DOI:** 10.1186/s43556-025-00321-3

**Published:** 2025-10-09

**Authors:** Daxin Cui, Xiaoqian Yu, Qiuyue Guan, Ying Shen, Jiajing Liao, Yin Liu, Zhiguang Su

**Affiliations:** 1 Center for High Altitude Medicine, State Key Laboratory of Biotherapy, West China Hospital, Sichuan University, Chengdu, China; 2 Department of Pain Management, West China Hospital, Sichuan University, Chengdu, China; 3 Department of Outpatient, Sichuan Provincial People’s Hospital, University of Electronic Science and Technology of China, Chengdu, China; 4 Department of Laboratory Medicine, West China Hospital, Sichuan University, Chengdu, China

**Keywords:** Cholesterol homeostasis, Metabolism regulation, Steroid hormone, Signal transduction, Cholesterol-related diseases, Cholesterol-lowering therapy

## Abstract

Cholesterol, an indispensable structural and signaling lipid, is fundamental to cellular membrane integrity, steroidogenesis, and developmental morphogen pathways. Its homeostasis hinges on the precise coordination of four interdependent metabolic modules: de novo biosynthesis, intestinal absorption, enzymatic conversion, and systemic clearance. This review delineates the molecular machinery governing these processes—from the Bloch/Kandutsch-Russell synthesis pathways and niemann-pick C1-like 1 (NPC1L1)-mediated cholesterol uptake to cholesterol 7α-hydroxylase (CYP7A1)-driven bile acid synthesis and HDL-dependent reverse transport. We further elucidate cholesterol’s multifaceted roles in lipid raft assembly, Hedgehog signal transduction, and vitamin D/hormone production. Critically, dysregulation of cholesterol flux underpins pathogenesis in atherosclerosis, metabolic dysfunction-associated fatty liver disease (MAFLD), neurodegenerative disorders, and oncogenesis, with disrupted synthesis, efflux, or esterification cascades serving as key drivers. Emerging therapeutic strategies extend beyond conventional statins and proprotein convertase subtilisin/kexin type 9 (PCSK9) inhibitors to include transformative modalities: CRISPR-based in vivo gene editing (e.g., VERVE-101 targeting *PCSK9*), small interfering RNA (siRNA) therapeutics (inclisiran), and microbiota-directed interventions. Pioneering approaches against targets Such as angiopoietin-like 3 (ANGPTL3), lipoprotein(a) [Lp(a)], and asialoglycoprotein receptor 1 (ASGR1)—alongside repurposed natural agents (berberine, probiotics)—offer promise for mitigating residual cardiovascular risk and advancing precision cardiometabolic medicine. By integrating mechanistic insights with clinical advancements, this review underscores the transition from broad-spectrum therapies to personalized, multi-target regimens, offering a roadmap for mitigating cholesterol-related diseases in the era of genomic and metabolic medicine.

## Introduction

Cholesterol, a vital lipid molecule, plays a multifaceted role in maintaining cellular and exosomic integrity, hormone synthesis, and signal transduction [1, 2]. Its homeostasis is meticulously regulated through an intricate interplay of biosynthesis, intestinal absorption, metabolic conversion, and clearance mechanisms. Dysregulation of these processes underpins a spectrum of pathologies, including atherosclerosis, aging and age-related disease, MAFLD, neurodegenerative disorders, and cancer, positioning cholesterol metabolism as a cornerstone of metabolic health research [3–5].

Despite advances in understanding these pathways, challenges persist. Genetic disorders such as familial hypercholesterolemia (FH) and acquired conditions like MAFLD illustrate the clinical complexity of cholesterol dysregulation. Conventional therapies, including statins and PCSK9 inhibitors, have revolutionized cardiovascular risk management [6], yet limitations such as statin intolerance, residual risk, and incomplete efficacy underscore the need for novel strategies [7]. Emerging approaches—gene editing, RNA interference, and targeted modulation of pathways like ANGPTL3 / liver X receptor (LXR) signaling—promise to redefine therapeutic paradigms.

This review comprehensively examines the molecular machinery of cholesterol metabolism, its physiological and pathological implications, and the evolution of therapeutic interventions. By integrating mechanistic insights with clinical advancements, we aim to elucidate current challenges and future directions in achieving precision lipid management and mitigating cholesterol-related diseases.

## Regulation of cholesterol homeostasis

**Fig. 1:**
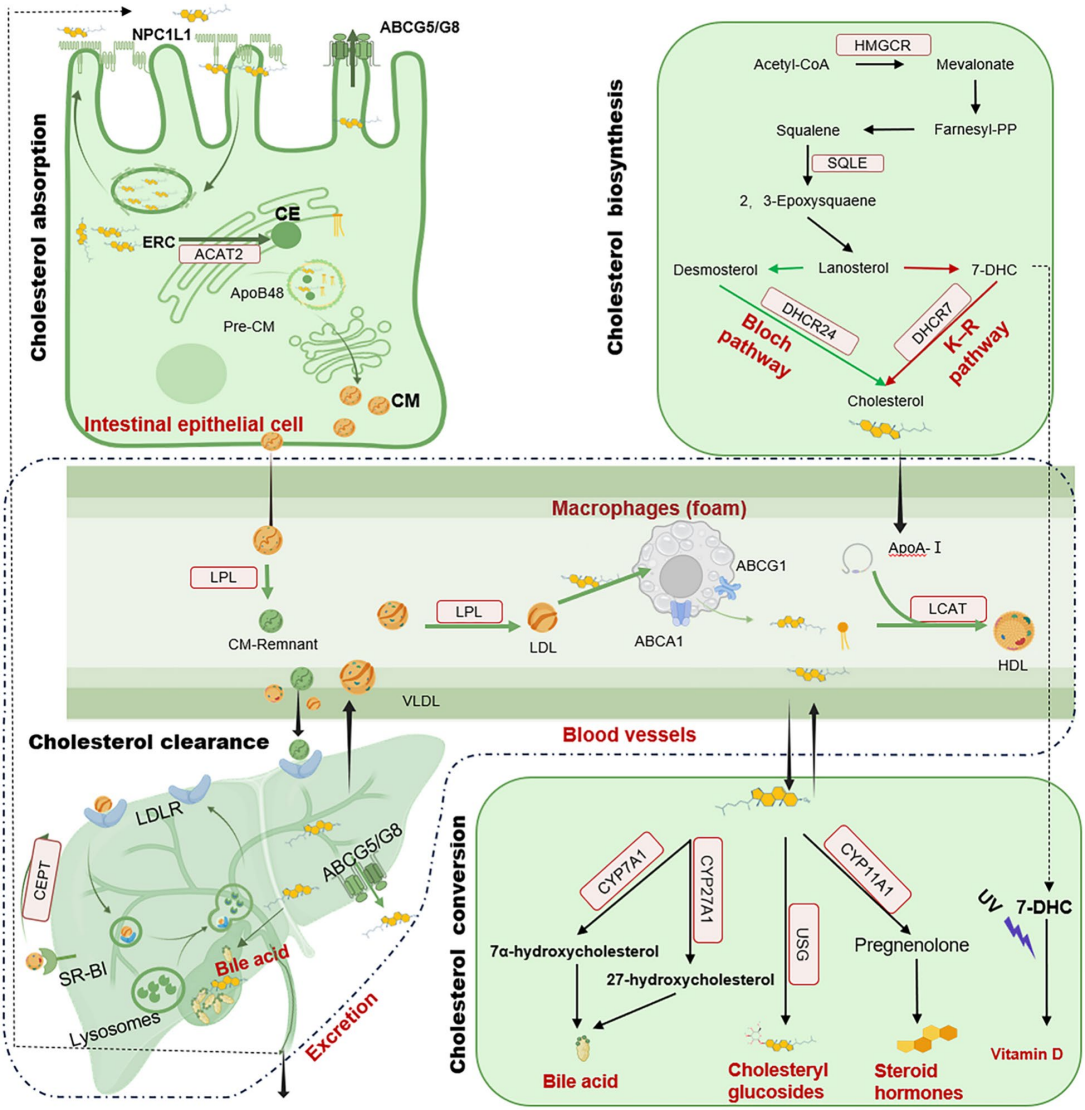
Intracellular metabolic pathways of cholesterol biosynthesis, absorption, and conversion. The de novo cholesterol biosynthetic pathway originates from acetyl-CoA and progressing through key intermediates such as HMG-CoA, mevalonate, Farnesyl-PP, and squalene, eventually forming cholesterol via lanosterol. Enzymes including HMGCR and SQLE are highlighted as critical rate-limiting steps. The downstream metabolic fates of cholesterol encompass its conversion into steroid hormones, bile acids, and vitamin D, as well as storage in lipid droplets as CE via ACAT, or transport in and out of cells through lipoprotein receptors (LDLR, SR-BI) and transporters (ABCA1, ABCG1, ABCG5/8). Cholesterol uptake from the intestinal lumen via NPC1L1 and its efflux to HDL particles are also depicted, Summarizing key intracellular cholesterol flux routes. Farnesyl-PP, farnesyl pyrophosphate; HMG-CoA, 3-hydroxy-3-methylglutaryl-CoA; HMGCR, HMG-CoA reductase; SQLE, squalene epoxidase; CE, cholesteryl esters; ACAT, acyl-CoA:cholesterol acyltransferase; SR-BI, scavenger receptor class B type I; NPC1L1, niemann-pick C1-like 1

Cholesterol homeostasis is meticulously orchestrated through the integration of four key metabolic modules: synthesis, absorption, conversion, and clearance (Fig. 1). This comprehensive metabolic cycle ensures that cholesterol fulfills its fundamental roles in membrane construction, signal transduction, and systemic homeostasis.

### Cholesterol biosynthesis

Vertebrate de novo cholesterol synthesis occurs predominantly in the liver (80%), with minor contributions from extrahepatic tissues (20%). This process is metabolically demanding, consuming 18 adenosine triphosphates (ATPs) and 16 nicotinamide adenine dinucleotides (NADs) phosphate reduced form (NADPH), as well as acetyl-CoA and oxygen, for each cholesterol molecule produced [8]. It proceeds through two evolutionarily conserved pathways: the canonical Bloch pathway (> 90% total output), elucidated by Konrad Bloch's Nobel-winning studies in the 1950s [9, 10], and the Kandutsch-Russell (K-R) pathway, discovered in the 1960s (Fig. 1). The K-R pathway is dominant under hypoxia or ultraviolet (UV) stress in tissues like skin and gonads [11]. Both pathways shared the same initial steps, beginning with the condensation of acetyl-CoA molecules. Thiolase combines two acetyl-CoA molecules to form acetoacetyl-CoA, to which 3-hydroxy-3-methylglutaryl-CoA (HMG-CoA) synthase adds a third acetyl-CoA, yielding HMG-CoA. The rate-limiting enzyme, HMG-CoA reductase (HMGCR), then reduces HMG-CoA to mevalonate (MVA) using NADPH. MVA undergoes sequential phosphorylation and isomerizations to produce farnesyl pyrophosphate (FPP). Squalene synthase (SQS) dimerizes FPP into squalene, which is subsequently oxidized by the second rate-limiting enzyme, squalene epoxidase (SQLE), to form 2,3-oxidosqualene. Lanosterol synthase (LSS) cyclizes 2,3-oxidosqualene into lanosterol, the first sterol intermediate. Beyond lanosterol, the Bloch pathway and K-R pathway diverge. In the Bloch pathway, lanosterol undergoes demethylation, desaturation, and reduction to ultimately yield cholesterol. In K-R pathway, lanosterol is sequentially converted into 24,25-dihydrolanosterol and 7-dehydrocholesterol (7-DHC), followed by reduction of 7-DHC to cholesterol via 7-DHC reductase (DHCR7). Although less efficient, the K-R pathway avoids oxygen-dependent steps, providing a physiological advantage in oxygen-limited environments.

### Cholesterol absorption

Cholesterol absorption, occurring primarily in the duodenum and proximal jejunum, is a tightly regulated process critical for maintaining systemic lipid homeostasis, ensuring efficient uptake of dietary and biliary cholesterol while preventing excessive accumulation. The central mediator of intestinal cholesterol absorption is the enterocyte membrane protein niemann-pick C1-like 1 (NPC1L1), which shuttles between the cell surface and endocytic recycling compartments (ERCs) to facilitate uptake. NPC1L1 contains five transmembrane domains, including a sterol-sensing domain that detects cholesterol levels [12]. High luminal cholesterol prompts free cholesterol integration into the enterocyte membrane, where NPC1L1 binds it and facilitates internalization. This occurs primarily via clathrin/AP2-mediated endocytosis, transporting cholesterol along actin filaments to ERCs for storage [13]. Cryo-electron microscopy studies reveal an additional mechanism, cholesterol binding induces a conformational change in NPC1L1, forming a transmembrane transport tunnel that directly facilitates uptake independent of endocytosis [14]. Under low-cholesterol conditions, NPC1L1 recycles back to the membrane to resume absorption [13].

Once internalized, cholesterol undergoes esterification or efflux to balance cellular levels. Free cholesterol traffics to the endoplasmic reticulum, where acyl-CoA:cholesterol acyltransferase 2 (ACAT2) esterifies it with fatty acids into cholesterol esters [15]. These hydrophobic esters are packaged into chylomicrons, transported to the Golgi apparatus for processing, and ultimately entering systemic circulation via the lymphatic system (thoracic duct) for delivery to peripheral tissues. Meanwhile, excess free cholesterol is actively pumped back into the intestinal lumen by the heterodimeric ATP-binding cassette (ABC) transporters G5/G8 (ABCG5/G8) [16]. This efflux mechanism critically limits net absorption and protects against cellular overload.

### Cholesterol conversion

Within cells, cholesterol serves as a versatile precursor for synthesizing biologically essential compounds [17], including bile acids, cholesteryl glucosides, vitamin D and various steroid hormones such as androgens, estrogens, progesterone, glucocorticoids, and mineralocorticoids.

#### Bile acid synthesis

In the liver, the majority of cholesterol is converted into bile acids, consisting mainly of primary bile acids like cholic acid (CA) and chenodeoxycholic acid (CDCA), along with secondary bile acids such as deoxycholic acid (DCA) and trace amounts of lithocholic acid (LCA), through two distinct pathways [18]. The classic pathway, responsible for over 90% of bile acid production, initiates with cholesterol 7α-hydroxylase (CYP7A1) hydroxylating cholesterol at the 7α-position. This rate-limiting step yields 7α-hydroxycholesterol, which is then converted to 7α-hydroxy-4-cholesten-3-one (C4) by 3β-hydroxy-Δ5-C27-steroid dehydrogenase (3β-HSD). Notably, C4 serves as a common precursor for both CA and CDCA and is often used as a serum biomarker for bile acid synthesis rate [18]. Conversely, the alternative pathway acts as an essential compensatory mechanism during the classic pathway impairment or metabolic stress, it begins with mitochondrial sterol 27-hydroxylase (CYP27A1) converting cholesterol to 27-hydroxycholesterol (27-OHC), followed by oxysterol 7α-hydroxylase (CYP7B1) catalyzing further hydroxylation to generate 3β,7α-dihydroxy-5-cholestenoic acid, which undergoes side-chain oxidation and shortening via 3β-HSD type 7 (HSD3B7) to produce CDCA. Although this alternative route accounts for only about 10% of bile acid synthesis under normal conditions, it becomes critically important in pathological states [19].

#### Cholesteryl glucosides (CGs)

CGs are sterol glycosides in which a glucose moiety is esterified to cholesterol’s hydroxyl group. CGs contribute to membrane stability by altering lipid packing and fluidity, particularly in lipid rafts [20]. CGs may also act as signaling molecules in immune responses. For instance, they are implicated in macrophage activation and cytokine production. In plants, CGs defend against microbial pathogens, suggesting analogous roles in mammalian innate immunity. In mammals, CGs are synthesized via enzymatic glycosylation of cholesterol primarily in the Golgi apparatus or endoplasmic reticulum, where cholesterol and UDP-glucose are accessible. UDP-glucose:sterol glucosyltransferase (USG) catalyzes the transfer of glucose from UDP-glucose to cholesterol’s 3β-hydroxyl group. CG production is regulated by cholesterol availability and cellular stress, under conditions of cholesterol overload or oxidative stress, CG synthesis may increase to modulate membrane fluidity or sequester excess cholesterol. Dysregulated CG synthesis is linked to lipid storage disorders and inflammatory diseases. For example, elevated CG levels are observed in Niemann-Pick type C disease, a lysosomal storage disorder. Interestingly, certain pathogenic organisms, such as *Helicobacter pylori*, are also capable of synthesizing cholesterol glucosides, although their biosynthetic pathways differ fundamentally from those in mammals [21].

#### Vitamin D synthesis

Vitamin D, a secosteroid critical for calcium homeostasis and immune regulation, is synthesized from 7-DHC through an UV-dependent pathway and subsequent enzymatic modifications [22]. In the epidermal stratum basal and spinosum, 7-DHC, an intermediate in the synthesis of cholesterol, is converted to pre-vitamin D3 upon exposure to UVB radiation (290–315 nm). This non-enzymatic reaction occurs spontaneously. Pre-vitamin D3 undergoes temperature-dependent isomerization to cholecalciferol (vitamin D3) over ~ 48 h. Vitamin D3 is hydroxylated in two sequential steps to achieve biological activity. In the liver, cytochrome P450 2R1 (CYP2R1) converts vitamin D3 to 25-hydroxyvitamin D3 [25(OH)D3], the major circulating form. In the kidney, cytochrome P450 27B1 (CYP27B1) hydroxylates 25(OH)D3 to 1,25-dihydroxyvitamin D3 [1,25(OH)2D3], which enhances intestinal absorption of calcium and phosphate and promotes renal reabsorption.

#### Steroid hormones

As the universal precursor of all steroid hormones, including mineralocorticoids, glucocorticoids, and sex hormones, cholesterol undergoes tissue-specific biotransformation within the adrenal cortex. This primary synthesis site is organized into three zones: the zona glomerulosa, which produces mineralocorticoids (e.g., aldosterone) to regulate electrolyte balance and blood pressure; the zona fasciculata, which synthesizes glucocorticoids (e.g., cortisol) governing stress response and metabolism; and the zona reticularis, which generates sex steroids (e.g., androgens) essential for reproductive physiology. For detailed biosynthetic pathways of these steroid classes, refer to Sect. " Cholesterol as the universal precursor to steroid hormone synthesis".

### Cholesterol clearance

Cholesterol homeostasis in mammals relies on tightly regulated clearance mechanisms to prevent pathological accumulation in tissues. Two major lipoprotein-mediated complementary pathways, including high-density lipoprotein (HDL)-driven reverse cholesterol transport (RCT) and low-density lipoprotein (LDL)-dependent hepatic uptake, are essential for maintaining cholesterol homeostasis [23]. HDL removes excess cholesterol from peripheral tissues, while LDL and its receptor LDLR ensures efficient hepatic uptake of circulating cholesterol. Therapeutic strategies targeting these pathways, such as LDLR- or HDL-boosting agents, hold promise for treating dyslipidemia and atherosclerosis.

#### HDL-mediated RCT

RCT is a critical process through which cholesterol is transported from peripheral tissues to the liver for excretion via bile or feces. A major component of the RCT pathway is HDL. The nascent HDL particles are discoidal in shape, composed of apolipoprotein A1 (ApoA1) and phospholipids [24]. HDL particles acquire free cholesterol from peripheral foam cells, such as macrophages or vascular smooth muscle cells, via the ABC transporters A1 (ABCA1) and G1 (ABCG1). ABCA1 interacts with lipid-poor apoA1, while ABCG1 facilitates cholesterol efflux to mature HDL [25]. Lecithin-cholesterol acyltransferase (LCAT) esterifies free cholesterol within HDL, converting it into cholesterol esters. This hydrophobic core transforms HDL into spherical particles. Mature HDL delivers cholesterol to the liver through scavenger receptor class B type I (SR-BI)-mediated selective uptake or cholesterol ester transfer protein (CETP)**-**facilitated transfer. SR-BI selectively extracts cholesterol esters from HDL without degrading the entire particle, CETP shuttles cholesterol esters from HDL to apolipoprotein B (ApoB)-containing lipoproteins (e.g., LDL, very-low-density lipoprotein (VLDL)), which are later cleared via hepatic LDLR [26].

#### LDL-mediated cholesterol clearance

LDL particles, carrying cholesterol esters and apolipoprotein B-100 (ApoB-100), are cleared predominantly by the liver. LDLRs, highly expressed in hepatocytes, binds ApoB-100 on LDL particles via electrostatic interactions. LDL-LDLR complexes are internalized into clathrin-coated vesicles, which fuse with lysosomes. Within lysosomes, cholesterol esters are hydrolyzed to free cholesterol, while LDLR is recycled to the cell surface. In some cases, HDL particles expressing apolipoprotein E (ApoE) can bind LDLR or LDL receptor-related protein 1 (LRP1), enabling their hepatic internalization and degradation. This pathway provides an auxiliary route for HDL cholesterol clearance [26].

Proprotein convertase subtilisin/kexin type 9 (PCSK9) is a member of the serine protease family known for its critical role in proteolytic activation, modification, and degradation of secreted proteins [27]. Hepatic LDLRs serve as receptors for circulating PCSK9. PCSK9 binds to the epidermal growth factor-like repeat A (EGF-A) domain of LDLRs via its catalytic domain, promoting LDLR endocytosis into endosomes. The acidic pH within endosomes enhances the PCSK9–LDLR interaction by 150-fold, preventing LDLR recycling. Consequently, the PCSK9–LDLR complex is directed to lysosomal degradation, reducing LDLR density on the hepatocyte surface, thereby decreasing hepatic LDL cholesterol (LDL-C) particle clearance and increasing circulating LDL-C levels [28]. Inhibiting PCSK9 enhances LDL-C clearance, a therapeutic strategy for hypercholesterolemia.

## Molecular mechanisms of cholesterol metabolism

**Fig. 2:**
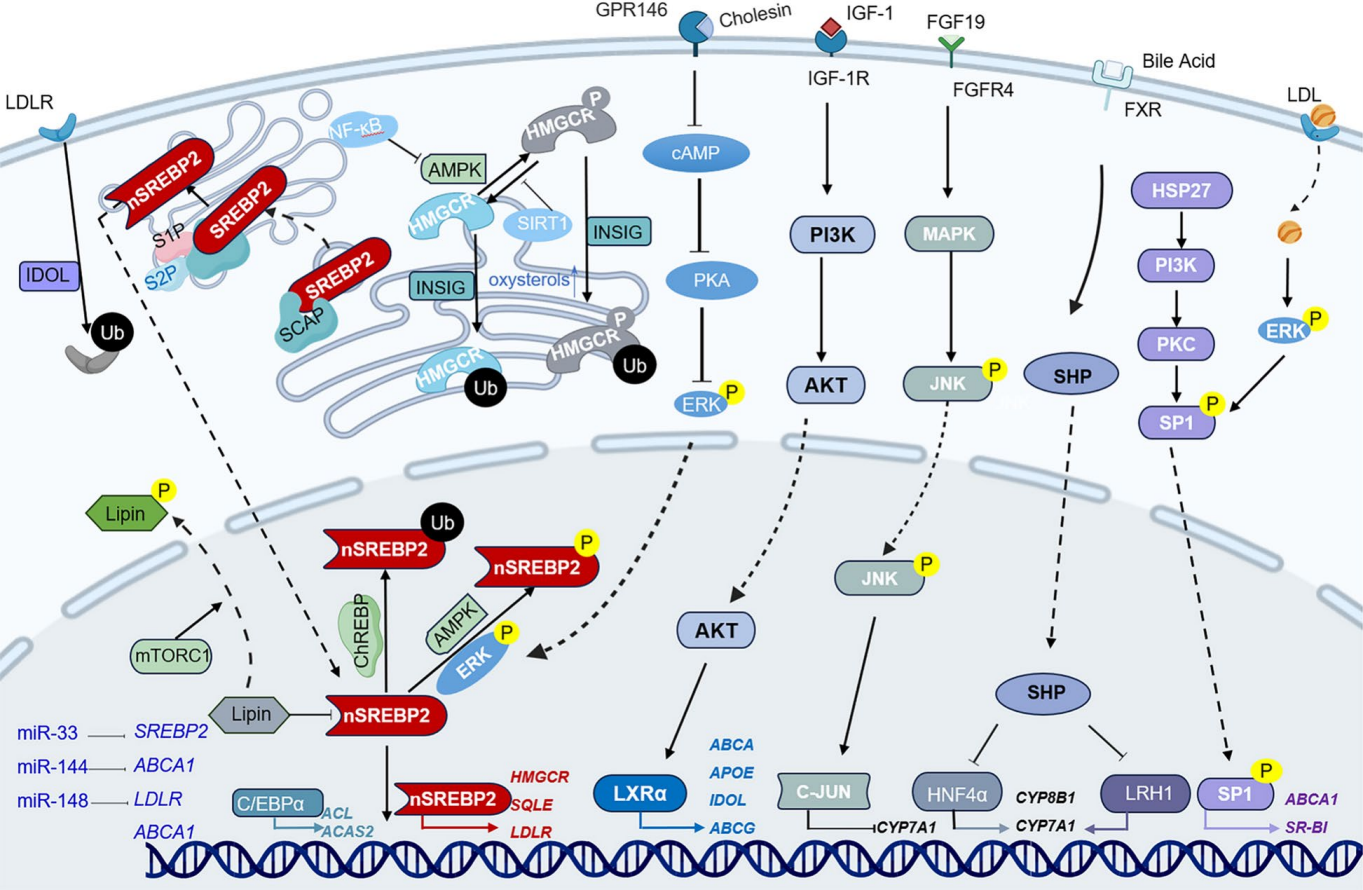
Regulatory network controlling cholesterol homeostasis. Intracellular cholesterol balance is maintained through the integration of signal transduction pathways with transcriptional and post-transcriptional mechanisms. Metabolic and hormonal cues, such as oxysterols, IGF-1, FGF19, activate or inhibit kinase cascades, including AMPK, ERK, JNK, PI3K/AKT, and cAMP/PKA. These pathways, in turn, regulate transcription factors and cholesterol-handling proteins. Key inhibitory regulators, such as IDOL and PCSK9, mediate LDL receptor degradation, modulating cholesterol uptake. Therapeutic agents like metformin and O-304 influence this network primarily through AMPK activation and IDOL suppression. Central to this network are key transcription factors, including nuclear SREBP2, C/EBPα/β, PPARα, which coordinate the expression of genes involved in cholesterol biosynthesis (e.g., HMGCR, HMGCS1), uptake (e.g., LDLR), efflux (e.g., ABCA1, ABCG1), and bile acid synthesis (e.g., CYP7A1). SREBP2 serves as a central node, with its activity modulated by oxysterol-mediated feedback and upstream signaling cascades. Post-transcriptional regulation is mediated by microRNAs including miR-33, miR-144, miR-148, miR-20a/b, and miR-34a, which suppress the translation of key mRNAs involved in cholesterol transport and metabolism. Additional modulators such as cholesin, HSP27, and epigenetic marks like histone acetylation, further influence gene expression profiles

Cholesterol metabolism is a tightly regulated process essential for cellular homeostasis, membrane integrity, and hormone synthesis. Dysregulation of cholesterol balance contributes to metabolic disorders, cardiovascular diseases, and liver pathologies. We explore the molecular machinery governing cholesterol biosynthesis, conversion, and clearance, focusing on key enzymes, transcription factors, and signaling pathways (Fig. 2). This comprehensive understanding of cholesterol metabolism regulation highlights the intricate mechanisms governing systemic cholesterol homeostasis.

### Key proteins and enzymes

#### HMGCR

HMGCR, the rate-limiting enzyme in cholesterol biosynthesis, catalyzes the conversion of HMG-CoA to mevalonate. Its structure comprises an N-terminal transmembrane domain and a hydrophilic C-terminal domain that extends into the cytosol to perform catalytic functions [29]. Four HMGCR monomers assemble into a tetramer to execute its catalytic function. As the critical enzyme in cholesterol biosynthesis, HMGCR is tightly regulated at transcriptional, translational, and post-translational levels.

Sterols, particularly oxysterols Like 25- or 27- hydroxycholesterol and methylated sterols Such as lanosterol and 24,25-dihydrolanosterol, induce HMGCR ubiquitination and Subsequent proteasomal degradation. In contrast, when sterol concentrations are low, sterol regulatory element-binding protein 2 (SREBP2) activates *HMGCR* gene transcription. However, translation of HMGCR mRNA can be inhibited by poorly characterized non-sterol isoprenoid receptors. Under sterol-depleted conditions, HMGCR remains relatively stable, with a half-life of approximately 12 h in cultured human fibroblasts [30, 31]. Members of the vitamin E family, δ-tocotrienol and γ-tocotrienol, also promote HMGCR degradation. Compared to sterols, cholesterol itself is a relatively weak inducer of HMGCR degradation. AMP-activated protein kinase (AMPK) phosphorylates HMGCR at serine 871 in rodents or Ser872 humans, located near the catalytic domain [32]. This phosphorylation inactivates HMGCR without affecting its sterol-induced degradation. Additionally, miR-34a, which is overexpressed in MAFLD, Suppresses sirtuin 1 (SIRT1), leading to AMPK dephosphorylation and subsequent HMGCR activation. This contributes to cholesterol accumulation in MAFLD [9, 33].

#### CYP7A1

CYP7A1, the rate-limiting enzyme in the classic bile acid synthesis, converts > 50% of bodily cholesterol in into bile acids. During RCT in peripheral tissues, peripheral cholesterol returns to the liver where CYP7A1 primarily mediates its conversion to bile acids before fecal excretion [34]. In rodents, a high-cholesterol diet activates LXRα, which induces *Cyp7a1* transcription via LXR response elements (LXREs), promoting bile acid synthesis. However, the human *CYP7A1* promoter lacks functional LXREs, rendering LXRα-*CYP7A1* signaling ineffective. Conversely, the farnesoid X receptor (FXR) pathway is conserved across species. FXR functions as a negative regulator of cholesterol metabolism by suppressing *CYP7A1* transcription [35]. Inhibition of FXR signaling upregulates CYP7A1 expression, thereby enhancing bile acid synthesis and maintaining cholesterol homeostasis. Additionally, *CYP7A1* transcription is negatively regulated by intestine-derived fibroblast growth factor 15 (FGF15), which binds hepatic FGFR4/β-Klotho, triggering a JNK-dependent intracellular signaling cascade that suppresses *CYP7A1* transcription [36]. Furthermore, CYP7A1 expression is subject to circadian rhythm regulation, with expression levels peaking around midday in mice, although feeding and fasting states also influence its expression levels [37].

#### CYP11A1

CYP11A1, also known as cholesterol side-chain cleavage enzyme (P450scc) located in the mitochondrial inner membrane of steroidogenic tissues (e.g., adrenal glands, gonads, and placenta), is a pivotal enzyme in steroidogenesis. It catalyzes the initial and rate-limiting step of cholesterol metabolism, converting cholesterol to pregnenolone, the universal precursor for all steroid hormones, such as glucocorticoids, mineralocorticoids, and sex hormones. CYP11 transcription is regulated by tissue-specific and hormone-responsive elements, and it can be induced by second messenger systems such as protein kinase A (PKA) and protein kinase C (PKC) [38]. The expression of CYP11A1 and other steroidogenic enzymes in adrenal glands and gonads requires the action of steroidogenic factor 1 (SF1), an orphan nuclear receptor [39]. In contrast, placental expression of *CYP11A1* is constitutive and independent of SF1, but it involves transcription factors from the CP2 (grainyhead) family, also known as LBP proteins, and TreP-132 [40, 41]. Long-term cellular stimulation over several days increases CYP11A1 levels and enhances steroidogenic capacity, leading to elevated basal steroid hormone production.

#### Apolipoprotein

Apolipoproteins (Apos), the protein constituents of lipoproteins, serve as critical regulators of cholesterol metabolism through their roles in lipid transport, enzymatic activation, and receptor-mediated signaling. Their dysfunction is closely associated with metabolic disturbances and heightened cardiovascular risks [42].

ApoA1, the major structural protein of HDL, is central to RCT [43]. It activates LCAT, esterifying free cholesterol for efficient packaging into HDL particles. ApoA1 also interacts with ABCA1 on macrophages to promote cholesterol efflux from peripheral tissues to HDL. Genetic deficiencies in *ApoA1* result in low HDL levels and accelerated atherosclerosis. Therapeutic strategies, such as reconstituted HDL infusions or ApoA1 mimetic peptides, aim to enhance RCT and reduce cardiovascular disease (CVD) risk [42].

ApoB exists in two forms, ApoB-48 and ApoB-100. ApoB-48 is synthesized in the intestine and serves as a crucial structural protein for chylomicrons. In contrast, ApoB-100 acts as the main structural component of VLDL, intermediate-density lipoprotein (IDL), LDL, and Lp(a). It plays an essential role in the assembly and secretion of VLDL in the liver and mediates cholesterol uptake in in peripheral tissues by binding to LDLRs. Elevated ApoB-containing lipoproteins, particularly LDL, are a hallmark of hypercholesterolemia [44]. Mutations in *ApoB* or *LDLR* (e.g., familial hypercholesterolemia) impair LDL clearance, leading to premature CVD. PCSK9 inhibitors, which stabilize LDLR expression, and siRNA therapies targeting ApoB synthesis (e.g., inclisiran) are emerging treatments.

ApoE is involved in the clearance of lipoprotein remnants from the bloodstream and is primarily found in HDL and remnant particles. It facilitates the recognition and uptake of lipoproteins by hepatic receptors, thereby regulating cholesterol metabolism. The *ApoE* gene exhibits three major isoforms, including ε2, ε3, and ε4, with ε4 allele carriers showing increased susceptibility to both Alzheimer's disease and CVD [45]. Notably, the ε2 variant impairs receptor binding, causing type III hyperlipoproteinemia. Beyond lipid metabolism, ApoE modulates neuroinflammation and amyloid-β clearance, underscoring its systemic regulatory roles.

Recent studies highlight additional Apos, such as ApoO and ApoF. ApoO modulates cholesterol metabolism by influencing bile acid and fecal cholesterol excretion [46]. It also impacts mitochondrial function, indirectly affecting lipid metabolism and obesity. ApoF acts as a natural CETP inhibitor, regulating the redistribution of cholesterol esters between HDL and LDL/VLDL [47]. This regulatory mechanism preserves HDL's atheroprotective properties while limiting LDL-C accumulation.

### Transcription factors

Cholesterol homeostasis is a dynamic process orchestrated by an intricate network of transcriptional regulators that adapt to cellular demands. At the core of this regulatory network lie SREBP2 and LXR, which balance cholesterol biosynthesis and efflux. Beyond these central players, peroxisome proliferator-activated receptors (PPARs) and auxiliary transcription factors such as HNF4α, SP1, C/EBP, and NF-κB further fine-tune cholesterol metabolism through tissue-specific and context-dependent mechanisms [48, 49].

#### SREBP2

SREBP2, anchored in the endoplasmic reticulum, is a transcription factor with a unique dual-domain structure. Its NH2-terminal domain contains DNA-binding motif and transcriptional activation domain, while the COOH-terminal regulatory domain interacts with SREBP cleavage-activating protein (SCAP). Under sterol-depleted conditions, the SCAP-SREBP complex dissociates from insulin-induced genes (INSIGs), leading to its ubiquitination and subsequent proteasomal degradation. The complex is then packaged into coat protein II (COPII)-coated vesicles, a process mediated by the small GTPase Sar1. COPII vesicles are assembled through the sequential recruitment of coat proteins, such as Sec23/Sec24 and Sec13/Sec31, and escort the SCAP-SREBP complex from the ER to the Golgi apparatus. At the Golgi, SREBP is cleaved sequentially by site-1 protease (S1P) and site-2 protease (S2P), releasing the transcriptionally active N-terminal fragment nSREBP2 [48]. The cleaved nSREBP2 translocates to the nucleus and activates genes containing sterol regulatory elements (SREs). The promoter regions of genes encoding key enzymes in the cholesterol biosynthesis pathway, such as *HMGCS*, *HMGCR*, *farnesyl diphosphate synthase (FDPS)*, and *SQS*, contain SREs. nSREBP2 binds to these SREs to promote gene transcription, thereby enhancing cholesterol synthesis [49]. Newly synthesized cholesterol is rapidly transferred from ER to the plasma membrane, and ER cholesterol levels directly influence overall cellular cholesterol balance. nSREBP2 not only induces genes involved in cholesterol synthesis and uptake but also inhibits *ABCA1* transcription, thereby suppressing cholesterol efflux [50, 51].Additionally, miR-33, a microRNA located within an intron of the SREBF2 locus, is co-transcribed with SREBF2 and acts to suppress cholesterol transport and export, rapidly restoring intracellular cholesterol concentrations [52]. When intracellular cholesterol levels increase, the SCAP binds INSIG proteins, blocking the transport of the SREBP2-SCAP complex from the ER to the Golgi apparatus and triggering ubiquitin-dependent proteasomal degradation of HMGCR via the INSIG/GRP78 pathway, reducing cholesterol synthesis. These mechanisms emphasize the feedback regulation of cholesterol synthesis based on intracellular cholesterol levels [53].

Emerging studies reveal that overexpression of long non-coding RNA lncGSAR in ovine ovarian granulosa cells promotes cell proliferation and estrogen secretion while suppressing progesterone production, with lncGSAR knockdown exhibiting opposing phenotypes. Mechanistically, lncGSAR functions as a competing endogenous RNA (ceRNA) to activate the SCAP/SREBP pathway by sequestering miR-125b [54]. Furthermore, palmitoyltransferase ZDHHC3 and depalmitoylase ABHD17A modulate cholesterol biosynthesis in hepatocellular carcinoma cells through dynamic S-acylation of SCAP at C264. ZDHHC3-mediated SCAP S-acylation antagonizes HACE1-catalyzed SCAP ubiquitination, while SREBP2 transcriptionally activates ZDHHC3 expression, establishing a positive feedback regulatory loop [55].

During luteinizing hormone (LH)-induced steroidogenesis via the cAMP-PKA signaling axis, SCAP enhances progesterone synthesis through dual inhibition of phosphodiesterases PDE4/PDE8. This involves elevated SCAP phosphorylation, which activates SREBP2 and upregulates cholesterol biosynthetic gene expression, these effects are completely abolished by SCAP knockout [56]. AMPK phosphorylates SREBP-1 at Ser372, inhibiting its proteolytic processing and nuclear translocation, thereby negatively regulating lipid synthesis, as seen with baicalein and coniferyl aldehyde through AMPK activation. Conversely, the insulin/AKT/mTORC1 axis promotes SREBP maturation and nuclear translocation, enhancing lipogenesis. Constitutive mTORC1 activation in tumor cells leads to persistent SREBP signaling. Elevated cholesterol levels inhibit SREBP activation through the formation of SCAP-INSIG complex, establishing negative feedback regulation in cholesterol homeostasis [56].

#### LXR

LXR is another key transcription factor that regulates cholesterol metabolism in conjunction with SREBP2, playing a significant role in cholesterol transport. When intracellular cholesterol levels rise, various oxysterols are generated through its metabolism, several of which serve as endogenous ligands to activate LXR signaling pathway. For example, cholesterol is hydroxylated at the C25 position to form 25-hydroxycholesterol (25-OHC) by the 25-hydroxylase (CH25H). Subsequently, 25-OHC is metabolized by CYP7B1 into 7α,25-dihydroxycholesterol (7α25-HC) [57]. These cholesterol metabolites, acting as ligands, bind to LXR, causing a conformational change in the LXR-retinoid X receptor (RXR) complex and releasing the co-repressor NCoR. The LXR-RXR complex then recruits co-activators such as histone acetyltransferase p300 and steroid receptor coactivator-2 (SRC-2), inducing the expression of downstream target genes, including ApoE, ABCA, ABCG, and the inducible degrader of LDLR (IDOL). Activation of ABC transporters and ApoE is crucial for clearing or recycling excess cholesterol in the central nervous system [58]. Furthermore, LXR directly targets the promoter of IDOL, an E3 ubiquitin ligase that promotes the endocytosis and degradation of LDLR, reducing cellular LDL uptake and lowering intracellular cholesterol levels. Overall, when intracellular cholesterol levels are elevated, LXR reduces cholesterol uptake and promotes efflux to lower cellular cholesterol levels [59]. Notably, under 25-OHC deficiency, the SREBP2 precursor undergoes proteolytic processing in the Golgi apparatus and translocates to the nucleus to activate sterol biosynthesis.

At the immune regulation level, intracellular cholesterol overload activates the cholesterol/LXR/CD38 cascade, inducing macrophage senescence through NAD + depletion [60]. During this process, LXR activation dually upregulates CD38 expression and enhances ABCA1/G1-mediated cholesterol efflux, suggesting that targeting the LXR/CD38/NAD + pathway may represent a novel strategy for cellular senescence intervention [61].

#### Peroxisome proliferator-activated receptors (PPARs)

PPARs, a family of ligand-activated nuclear receptors comprising three isoforms (PPARα, PPARγ, and PPARβ/δ), serve as master regulators of lipid homeostasis through their tissue-specific expression patterns and pleiotropic functions in bile acid metabolism, inflammatory modulation, and fibrotic processes. Emerging evidence positions PPARα and PPARγ as central players in cholesterol catabolism and transport, while PPARβ/δ demonstrates complementary regulatory effects through indirect pathways.

PPARα is a key regulator of hepatic cholesterol catabolism. It primarily acts by inhibiting bile acid synthesis, the Major pathway for cholesterol breakdown in the Liver. This is achieved through the downregulation of cholesterol 7α-hydroxylase (CYP7A1), the rate-limiting enzyme in bile acid biosynthesis. Additionally, PPARα enhances bile acid secretion by upregulating bile salt export pump (BSEP) and multidrug resistance-associated protein 2 (*MRP2*), reduces bile acid toxicity by increasing *MDR3* expression, and promotes bile acid detoxification by inducing *CYP3A4* [62]. Activation of PPARα, such as through bezafibrate, has been shown to reduce CYP7A1 activity by approximately 60% and lower cholesterol 7α-hydroxylation by 55%, underscoring its inhibitory role in bile acid synthesis [63]. PPARα also facilitates RCT by upregulating HDL biogenesis genes, including *ApoA1*, *ApoA2*, *SR-BI*, and *ABCA1* [64]. This leads to an increase in plasma HDL-C levels by 5–15% and a reduction in coronary heart disease risk by approximately 25% [63]. Furthermore, PPARα enhances cholesterol efflux from macrophages by inhibiting CETP activity. In the intestine, PPARα reduces dietary cholesterol absorption by downregulating *NPC1L1*, a key transporter responsible for cholesterol uptake. This effect is abolished in *Pparα*-deficient mice, highlighting its specificity. PPARα agonists, such as gemfibrozil and fenofibrate, have demonstrated significant reductions in cholesterol absorption efficiency. Moreover, PPARα suppresses hepatic cholesterol synthesis by inhibiting SREBP-2 maturation and decreasing HMG-CoA reductase activity.

PPARγ also contributes to cholesterol metabolism by upregulating SR-BI in hepatocytes, facilitating the selective uptake of HDL cholesterol esters and enhancing RCT [65]. Additionally, PPARγ promotes cholesterol efflux in macrophages by inducing *ABCA1* and *ABCG1*, offering potential atheroprotective benefits [66, 67]. PPARγ agonists, such as troglitazone, have been shown to suppress de novo cholesterol synthesis in HepG2 and Caco-2 cells [68]. While less characterized, PPARβ/δ has also been implicated in cholesterol regulation as well. Agonists like GW610742 reduce intestinal cholesterol absorption by downregulating *NPC1L1* mRNA expression and lower LDL-C while elevate HDL levels. However, the precise molecular mechanisms underlying these effects remain to be fully elucidated [69].

#### Other transcriptional factors

Beyond the canonical regulators SREBP2, LXR, and PPARs, transcription factors Such as hepatocyte nuclear factor 4α (HNF4α), specificity protein 1 (SP1), CCAAT/enhancer-binding protein (C/EBP), and nuclear factor kappa B (NF-κB) also play significant roles in cholesterol metabolism through tissue-specific and context-dependent mechanisms.

HNF4α is a liver-enriched nuclear receptor that directly activates genes critical for cholesterol and bile acid metabolism, including *CYP7A1, SR-BI*, and *ApoB*) [70]. It binds to direct repeat 1 (DR1) motifs in target promoters and often cooperates with FXR to balance cholesterol catabolism [71]. HNF4α is suppressed by pro-inflammatory cytokines such as IL-1β, linking inflammation to impaired cholesterol clearance. Deletion of HNF4α significantly reduces the expression of *CYP7A1* and *CYP8B1*, which encode the rate-limiting and key enzymes for bile acid synthesis, respectively [70]. This results in a marked reduction in bile acid production.

SP1 orchestrates a complex regulatory network governing cholesterol metabolism through dynamic phosphorylation-mediated transcriptional modulation of downstream target genes. Phosphorylation of SP1 via the PI3K/PKC or ERK signaling pathways enhances its binding affinity to the *ABCA1* promoter, thereby facilitating cholesterol efflux from Macrophages. For instance, heat shock protein 27 activates the PI3K/PKC cascade to induce SP1 phosphorylation, while the R5-6 peptide augments SP1 promoter binding activity by similarly promoting its phosphorylation [72]. LDL stimulates SP1 phosphorylation at the Ser702 residue via the ERK pathway, recruiting the histone acetyltransferase p300 to the *SR-BI* promoter, upregulating *SR-BI* expression and promoting RCT. Elevated homocysteine levels, in contrast, suppress *SR-BI* expression by inhibiting SP1 binding and enhancing DNMT3b-mediated promoter methylation [73]. ERK1/2-mediated SP1 phosphorylation also increases its binding to GC-rich regions within the *LDLR* promoter, stabilizing transcription initiation complexes and enhancing *LDLR* transcription. For example, kaempferol utilizes this pathway to upregulate LDLR expression, facilitating plasma LDL clearance. SP1 synergizes with SREBP2 to amplify *HMGCR* expression under low-cholesterol conditions [5]. Hyperactivation of SP1 in diabetes exacerbates hepatic cholesterol accumulation, highlighting the SP1-centered regulatory network as a promising therapeutic target for diabetic dyslipidemia [74].

C/EBP family comprises several members, including C/EBPα, C/EBPβ, and C/EBPδ, exerting significant influence on lipid metabolism, particularly through interactions with SREBP family. They coordinately regulate the expression of lipogenic genes, such as ATP citrate lyase (ACL) and acetyl-CoA synthetase 2 (ACAS2), which are involved in generating precursors for cholesterol biosynthesis. This suggests that C/EBPs may indirectly affect cholesterol synthesis [75]. In addition, C/EBPs may bind to the promoter region of human HMGCR, the rate-limiting enzyme in cholesterol biosynthesis, directly regulate cholesterol synthesis at the transcriptional level [76]. Beyond cholesterol synthesis, C/EBPs also contribute to the maintenance of intracellular cholesterol homeostasis. Deletion of C/EBPβ significantly reduces total cholesterol and LDL-cholesterol levels, while upregulating the expression of CYP7A1 and ATP-binding cassette transporter G1 (ABCG1). The former facilitates the conversion of cholesterol into bile acids, and the latter mediates cholesterol efflux to HDL. These findings suggest that C/EBPβ may repress the expression of these genes, thereby promoting intracellular cholesterol accumulation [77].

NF-κB is primarily activated during inflammation and drives metabolic dysregulation through modulation of expression of key enzymes in cholesterol biosynthesis. Chronic activation of NF-κB signaling inhibits AMPK phosphorylation, removing AMPK-mediated repression of cholesterol synthesis [78]. NF-κB also enhances the mevalonate pathway through two distinct mechanisms. On the one hand, NF-κB decreases HMGCR phosphorylation, leading to de-repression and increased enzymatic activity. On the other hand, NF-κB upregulates the expression of HMG-CoA synthase 1 (HMGCS1) protein, promoting the conversion of acetyl-CoA to mevalonate [33]. Furthermore, NF-κB indirectly disrupts cholesterol homeostasis by repressing CYP7A1 and ABCA1 via TNF-α/IL-6 signaling [10]. Additionally, NF-κB upregulates PCSK9, which degrades LDLR and elevates circulating LDL-C.

### Signaling pathways in cholesterol metabolism regulation

Cholesterol metabolism is a tightly regulated process involving numerous signaling pathways that coordinate cholesterol synthesis, transport, and elimination (Fig. 2). These pathways play crucial roles in maintaining lipid homeostasis and preventing metabolic disorders such as atherosclerosis and hypercholesterolemia.

#### IGFBPL1-LXRα axis enhancing RCT

Insulin-like growth factor (IGF)-binding protein-like 1 (IGFBPL1) strengthens IGF1 binding to its receptor IGF1R [79], amplifying PI3K/AKT signaling and promoting LXRα expression. As a key transcription factor, LXRα directly bind to the *ABCG1* promoter, upregulating its transcription. ABCG1 functions as a homodimer to mediate cholesterol efflux to HDL particles. Experimental data demonstrate that IGFBPL1 overexpression increases ABCG1 protein levels by 2.3-fold and enhances cholesterol efflux efficiency by 40% [80]. This mechanism significantly enhances RCT, reduces lipid accumulation in macrophages, and mitigates atherosclerotic plaque development. Notably, the inhibitory effect of IGFBPL1 on lipid accumulation is reversed by the IGF-1R antagonist picropodophyllin (PPP), while the LXRα antagonist SR9238 abrogates IGFBPL1 function and restores intracellular lipid deposition.

#### mTOR complex 1 (mTORC1) and nutrient sensing

mTORC1 is a primary regulator of anabolic processes. It increases nSREBP2 levels by phosphorylating and preventing the nuclear entry of Lipin 1 [81]. Conversely, the lipogenic transcription factor carbohydrate response element-binding protein (ChREBP) promotes nSREBP2 ubiquitination and proteasomal degradation through an unknown mechanism [82]. Fasting activates SIRT1-mediated deacetylation of SREBP2, halting the energy-consuming cholesterol biosynthesis process under nutrient-deprived conditions [83].In addition to acetylation, nSREBP2 can undergo phosphorylation by ERK and AMPK, leading to increased or decreased transcriptional activity, respectively. Sumoylation of nSREBP2 also reduces its transcriptional activity. AMPK has been identified as an upstream kinase of LXR. Activated AMPK (phosphorylated AMPK) inhibits the production of endogenous LXR ligands, thereby reducing LXR expression and blocking LXR-mediated transcriptional regulation [84].

#### Poly (ADP-ribose) polymerases (PARPs) multidimensionally regulate lipid metabolism

PARP family enzymes, including PARP1, PARP2, PARP3, tankyrases, PARP9, PARP10, and PARP14, play multifactorial roles in lipid metabolism [85]. Their activity is finely modulated by cholesterol-based compounds such as oxysterols, steroid hormones, and bile acids. These enzymes are implicated in diverse lipid-related processes, including lipotoxicity, fatty acid and steroid biosynthesis, lipoprotein homeostasis, and fatty acid oxidation [86]. Additionally, PARPs function as cofactors for lipid-sensitive nuclear receptors and transcription factors, influencing lipid metabolic pathways and maintaining lipid homeostasis. For example, PARPs may modulate cholesterol efflux and bile acid synthesis indirectly through interactions with LXRα or FXR [87].

#### FXR-FGF15/19-MAPK axis maintains bile acid homeostasis

The FXR-FGF15/19-MAPK axis constitutes a sophisticated interorgan signaling network that dynamically regulates bile acid synthesis. This axis integrates hepatic and intestinal signals to maintain cholesterol-bile acid metabolic balance. Bile acids activate hepatic FXR, which induces the small heterodimer partner (SHP) to inhibit the transcription of *Cyp7a1* and *Cyp8b1* in hepatocytes. Meanwhile, bile acid-activated intestinal FXR induces fibroblast growth factor 15 (FGF15, mouse)/FGF19 (human) secretion. Circulating FGF15/19 binds FGFR4/β-Klotho receptor complex on hepatocytes, triggering the MAPK cascade, specifically c-Jun, leading to the inhibition of *Cyp7a1* and *Cyp8b1* transcription via AP-1 site competition [88]. In human primary hepatocytes, bile acid-induced FGF19 activates the MAPK pathway through ERK1/2, suppressing *CYP7A1* transcription independently of SHP. Notably, hepatic FXR/SHP primarily inhibits *Cyp8b1*, while intestinal FXR/FGF15 preferentially inhibits *Cyp7a1* transcription. Recent studies implicate Diet1 protein in regulating bile acid synthesis. This protein, which consists of tandem low-density lipoprotein receptor and MAM (meprin-A5-protein tyrosine phosphatase mu) domains and is primarily expressed in small intestinal enterocytes, potentially enhances intestinal FGF15 production to modulate hepatic FGFR4 sensitivity [89].

#### Dual roles of cAMP/ protein kinase A (PKA) signaling pathway in cholesterol homeostasis

The cAMP/PKA pathway exerts temporally distinct and context-dependent effects on cholesterol metabolism, displaying a unique dual regulatory capacity. In primary human hepatocytes, cAMP signaling potently inhibits *CYP7A1* transcription, the rate-limiting enzyme in bile acid synthesis, suppressing cholesterol catabolism. Luciferase reporter assays demonstrate cAMP/PKA-mediated suppression of the human *CYP7A1* promoter, contrasting with its stimulatory effect on phosphoenolpyruvate carboxykinase (*PEPCK*). Glucagon-activated cAMP signaling increases HNF4α phosphorylation, reducing its chromatin occupancy at the *CYP7A1* locus and impairing transcriptional activation [90]. Paradoxically, cAMP/PKA signaling concurrently activates cholesterol biosynthesis and steroid hormone production. Through the cAMP/PKA pathway, SIRT1 expression is downregulated, leading to increased histone acetylation (H3K14ac and H3K27ac) and elevated expression of cholesterol biosynthetic genes, such as SREBF2, HMGCR, and HMGCS1. In addition, the cAMP/PKA signaling activate cholesteryl ester hydrolase (CEH) and StAR, facilitating the conversion of cholesteryl esters into free cholesterol and promoting cholesterol transport into mitochondria [91]. While the repression of *CYP7A1* by cAMP occurs rapidly, the stimulation of the expression of steroidogenic genes *CYP11A1* and *CYP17A1* is a longer-term effect that manifests over several hours, ensuring sustained production of steroid hormones [92]. This dual regulation enables coordinated suppression of cholesterol elimination via bile acids while prioritizing cholesterol utilization for acute and chronic steroid hormone production, illustrating the pathway's central role in metabolic adaptation to hormonal signals.

#### Cholesin and GPR146 mediate intestinal–hepatic cholesterol dialogue

Cholesin, a peptide highly expressed in gastrointestinal epithelial cells, is secreted via exosomes in response to cholesterol uptake mediated by NPC1L1. Both ezetimibe treatment and genetic deletion of *NPC1L1* inhibit intestinal cholesterol absorption, leading to a reduction in cholesin secretion [93]. GPR146, which shares a genetic locus with cholesin, is predominantly expressed in the liver. Depletion of GPR146 lowers circulating cholesterol and triglyceride levels and prevents atherosclerosis development in *Ldlr*-deficient mice. Upon activation, GPR146 couples with the Gαi protein, promoting cAMP production and activating PKA. This cascade subsequently triggers ERK1/2 phosphorylation, leading to the activation of SREBP2 and upregulation of cholesterol biosynthetic enzymes such as HMGCR [94]. Cholesin disrupts the interaction between GPR146 and Gαi, thereby reducing cAMP levels. This antagonizes the cAMP/PKA/ERK1/2 signaling pathway and downregulates hepatic cholesterol synthesis. Thus, the cholesin–GPR146 axis constitutes a key regulatory mechanism linking intestinal cholesterol sensing to hepatic cholesterol homeostasis [95].

#### AMPK

AMPK functions as a pivotal metabolic sensor, orchestrating multiple aspects of cholesterol metabolism. Pharmacological activation of AMPK using agents such as metformin or O-304 has been shown to markedly reduce hepatic cholesterol synthesis. One of its primary mechanisms involves the direct phosphorylation of HMGCR, the rate-limiting enzyme in cholesterol biosynthesis. Specifically, phosphorylation at the Ser871 residue leads to enzyme inactivation [96]. In addition to directly regulating HMGCR, AMPK also suppresses lipid synthesis at the transcriptional level by downregulating the mRNA expression of SREBP1 and reducing the protein level of hepatocyte nuclear factor 4 alpha (HNF-4α). These changes result in diminished transcription of lipogenic and cholesterogenic genes, further limiting substrate availability for cholesterol biosynthesis. This mechanism has been particularly noted in MAFLD models [84]. Furthermore, AMPK activation promotes RCT by upregulating ABCG1 expression in macrophages. This facilitates cholesterol efflux, reduces foam cell formation, and alleviates atherosclerotic burden [97].

## Biological functions of cholesterol

**Fig. 3:**
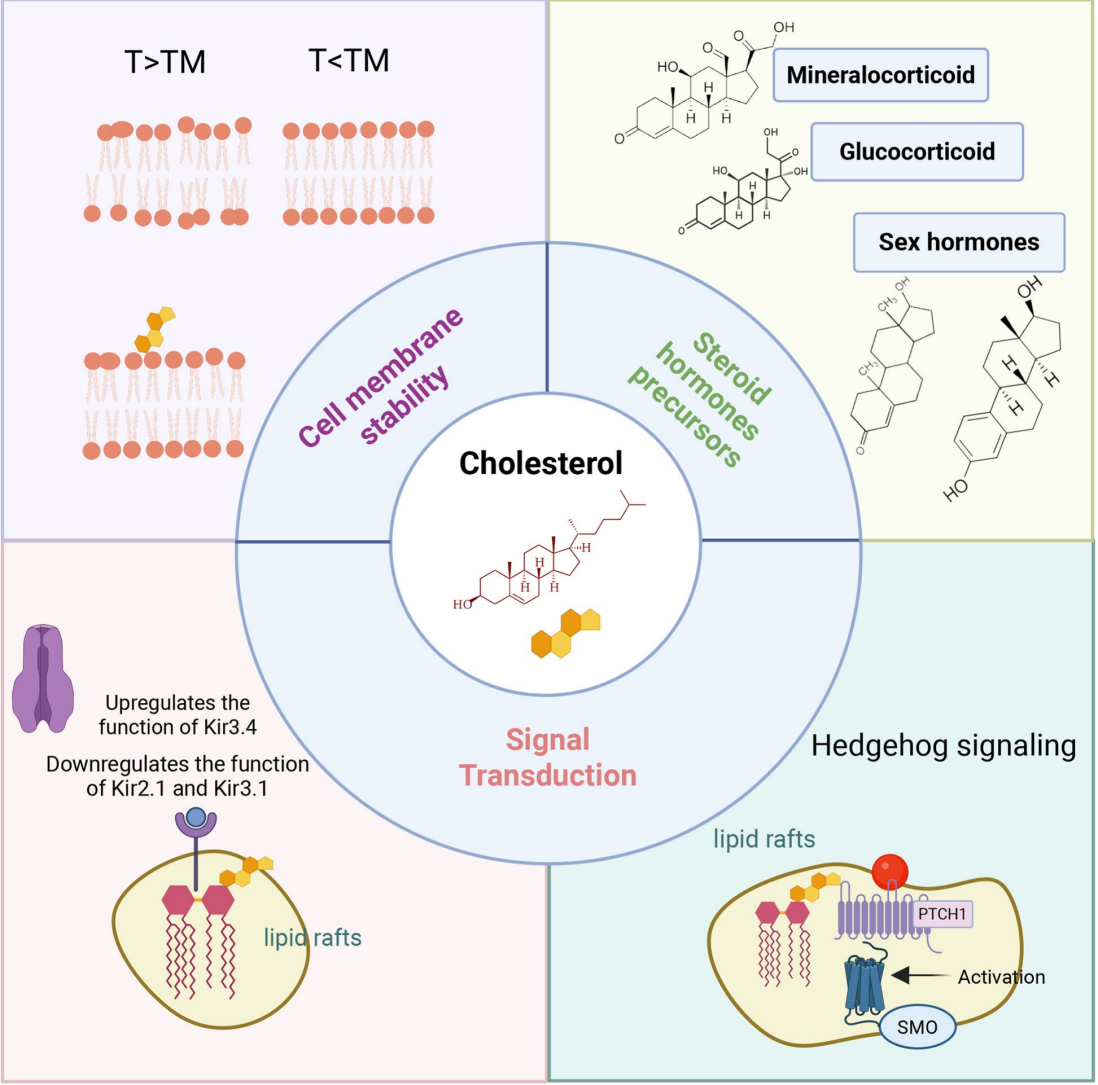
The biological roles of cholesterol. Cholesterol is a multifunctional molecule that plays essential roles in various biological processes. It is a key component of cell membrane, accounting for approximately 20%-30% of membrane lipids. Cholesterol is crucial for maintaining membrane integrity and fluidity. A pure phospholipid bilayer transitions from a gel (rigid) phase to a liquid crystalline (fluid) phase at its TM, and cholesterol dynamically modulates this transition to optimize membrane properties. Beyond its structural roles, cholesterol is a central regulator of Lipid metabolism and a precursor for essential hormones. It also modulates signal transduction pathways through multiple mechanisms. Cholesterol-rich microdomains, known as Lipid rafts, serve as platforms for the organization and activation of signaling molecules. For example, cholesterol directly interacts with the 12-transmembrane protein PTCH1, which structurally inhibits SMO. This interaction enables precise spatiotemporal control of the canonical Hedgehog signaling pathway, which is critical for development, immunity, and disease. Cholesterol also bidirectionally regulates ion channel function through both structural and allosteric mechanisms. Specifically, it upregulates the function of the Kir3.4 channel while downregulating the functions of Kir2.1 and Kir3.1 channels. These interactions highlight cholesterol’s diverse roles in cellular signaling and homeostasis. PTCH1, patched1; SMO, smoothened; TM, melting temperature. This figure was created via BioRender

Cholesterol is a multifunctional molecule with a wide range of biological roles. Its functions extend from maintaining membrane integrity and fluidity to serving as a precursor for essential hormones and bile acids. Additionally, cholesterol plays a crucial role in regulating lipid metabolism, modulating signal transduction pathways, and influencing immune function (Fig. 3). Understanding the diverse roles of cholesterol is essential for appreciating its importance in maintaining overall health and for developing therapeutic strategies to address cholesterol-related disorders.

### Cholesterol and cell membrane stability

Cholesterol is a key component of eukaryotic cell membranes, accounting for approximately 20%-30% of membrane lipids. A pure phospholipid bilayer exists in a gel (rigid) phase below its transition temperature (TM) and in a liquid crystalline (fluid) phase above Tₘ (Fig. 3). By intercalating among the phospholipid chains, cholesterol can smooth out the sharp inflection point of the phase transition and contribute to buffering the phase change, thereby stabilizing the liquid crystalline phase over a broader temperature range [98]. Thus, when the environment is near or above the transition temperature, cholesterol restricts the vigorous movements—especially lateral motions—of the phospholipid chains, thereby preventing excessive membrane fluidity. Conversely, at lower temperatures, the sterol ring of cholesterol hinders the orderly arrangement of phospholipid chains, preventing the formation of a rigid gel state [99].

Cholesterol's amphipathic architecture enables dual regulatory functions. On one hand, the hydrophobic sterol ring embeds within the hydrophobic tails of phospholipids, associating with saturated fatty acid chains to increase membrane density. By filling the gaps between phospholipid chains, cholesterol reduces membrane defects and thereby enhances mechanical strength [100]. On the other hand, the flexible portion of cholesterol’s alkyl chain can interact with the hydrophobic tails of unsaturated fatty acid chains, diminishing the disorderly packing caused by their kinks and preventing excessive fluidity [101].

Moreover, cholesterol collaborates with sphingolipids to form lipid raft domains, which are spatially segregated microdomains characterized by a high degree of order yet retain a certain level of fluidity, known as the liquid-ordered (Lo) phase. In contrast, the surrounding regions, rich in unsaturated phospholipids, exhibit high fluidity that facilitates substance diffusion and membrane deformation, and are known as the liquid-disordered (Ld) phase [102]. By regulating the assembly and disassembly of lipid rafts, cholesterol indirectly affects the fluidity of non-raft regions. Changes in the fluidity of these lipid rafts can also activate membrane proteins such as tyrosine receptor kinases, thereby influencing the efficiency of signal transduction [103].

### Cholesterol as a master regulator of cellular signal transduction

Cholesterol plays a dynamic role in orchestrating intracellular signaling by modulating membrane organization, receptor activation, and downstream effector functions. Beyond its classical role in membrane fluidity, cholesterol-rich microdomains (lipid rafts) and direct interactions with signaling molecules enable spatiotemporal control of diverse pathways, with implications for development, immunity, and disease [104].

#### Membrane microdomain organization and signaling efficiency

Lipid rafts, cholesterol-sphingomyelin-enriched membrane platforms, serve as activation hubs for receptors such as G protein-coupled receptors (GPCRs) and epidermal growth factor receptor. These microdomains spatially segregate signaling components to enhance specificity and efficiency. In the tyrosine kinase signaling pathway, the recruitment of adapter proteins, scaffolding proteins, and enzymes to the cytoplasmic side leads to ligand activation. The activated ligand can bind to an individual receptor and induce its activation. When receptor activation occurs within a lipid raft, the resulting signaling complex is protected from the influence of non-raft-associated enzymes; conversely, if activation occurs outside of lipid rafts, the signal transduction process may be adversely affected [105]. When lipid rafts are spatially segregated, they only coalesce following ligand binding and receptor activation, and such aggregation can establish a signaling platform [106].

In the immune system, lipid rafts on immune cell membranes dynamically assemble receptor proteins, ion channels, and effector enzyme complexes to form functional signaling units that regulate immune responses. In the immunological synapse formed between antigen-presenting cells and T cells via antigen presentation, structural evolution involves the spatial reorganization of lipid rafts: peripheral raft microdomains migrate centripetally and coalesce into a supramolecular activation cluster (SMAC). Notably, both this aggregation process and the functional activation of the T cell receptor depend on the unique liquid-ordered microdomains constructed by the cholesterol–sphingomyelin bilayer within lipid rafts. From a mechanistic regulatory perspective, the dynamic interplay between lipid rafts and the cytoskeletal network forms a key regulatory node in the T cell activation cascade, while the lateral migration and spatial aggregation of raft microdomains can significantly enhance signal transduction efficiency within the immunological synapse [107].

#### Ion channels modulation via cholesterol interactions

Cholesterol bidirectionally regulates ion channel function through structural and allosteric mechanisms. Alterations in cholesterol levels may affect ion channel function. For example, inwardly rectifying potassium (Kir) channels are known to be influenced by such change (Fig. 3). Specifically, cholesterol upregulates the function of Kir3.4 while downregulating the functions of Kir2.1 and Kir3.1. One contributing factor to these differences is the alteration in cholesterol distribution [108]. Compared to Kir2.1, the potential cholesterol binding site of Kir3.4 has undergone alterations. The preferential accumulation of cholesterol in the distal transmembrane helix is allosterically coupled with the conformational dynamics at the level of the selectivity filter. This allosteric coupling between channel function and lipid binding is a necessary mechanism for the PIP-mediated activation of Kir channels [109].

#### Cholesterol as a central hub in Hedgehog (HH) signaling

The evolutionarily conserved HH signaling cascade operates through canonical and non-canonical modalities to orchestrate developmental morphogenesis and tissue homeostasis [110]. Pathological disruption of this pathway manifests as multi-organ congenital malformations, underscoring its biological significance [111]. In the HH canonical pathway, HH ligand binding inactivates patched-1 (PTCH1), a 12-transmembrane domain receptor (Fig. 3). This relieves PTCH1-mediated suppression of smoothened (SMO), a GPCR with 7-transmembrane topology. Activated SMO initiates cytoplasmic signal transduction culminating in context-dependent activation of Ci/GLI transcription factors, thereby executing tissue-specific gene programs [112]. In ligand-absent conditions, PTCH1 maintains SMO inhibition, converting GLI proteins into transcriptional repressors that silence HH target genes. In the noncanonical signaling pathway, HH signaling can occur through non-Gli-dependent mechanisms or via direct activation of Gli in the absence of SMO or PTCH1 [113].

HH is intricately regulated by cholesterol at multiple levels. Cholesterol is essential for the biosynthesis of HH ligands, the generation of the signal, and the transduction of that signal from the cell surface to the intracellular compartment. Both SMO and PTCH1 are located within lipid raft microdomains, and depletion of plasma membrane cholesterol can alter the distribution of HH receptor complexes in cholesterol-enriched microdomains, thereby impacting HH signaling [114]. Moreover, defects in cholesterol synthesis that result in sterol depletion can adversely affect SMO activity, compromising the HH signaling response [115]. Additionally, SMO contains a sterol-sensing domain that binds cholesterol derivatives, modulating its conformational activation. Sterol synthesis defects, such as those seen in Smith-Lemli-Opitz syndrome, impair HH signaling and cause craniofacial anomalies and limb malformations [14, 20]. Recent studies suggest that cholesterol overload can hyperactivate HH signaling in chondrocytes, promoting cartilage degradation and the pathogenesis of osteoarthritis. Inhibition of HH signaling may offer therapeutic benefits for osteoarthritis patients, highlighting the potential of targeting the interaction between cholesterol and SMO for disease management [116]. Indeed, SMO inhibitors (e.g., vismodegib) have been shown to reduce ectopic ossification in preclinical models [21].

In addition, recent research has revealed that N1-methyladenosine (m6A) methylation in tRNA is significantly elevated in tumor tissues from patients with hepatocellular carcinoma. TRMT6 and TRMT61A form an m6A methyltransferase complex, and m6A methylation signaling is increased in liver cancer stem cells (CSCs). Mechanistically, TRMT6/TRMT61A-mediated m6A methylation in tRNA enhances the translation of PPARδ, which in turn triggers cholesterol synthesis to activate the HH signaling pathway, fueling CSC self-renewal and tumorigenesis. Thus, inhibiting the binding of cholesterol to SMO could block CSC propagation and represents a potential therapeutic strategy for liver cancer [117].

### Cholesterol as the universal precursor to steroid hormone synthesis

**Fig. 4:**
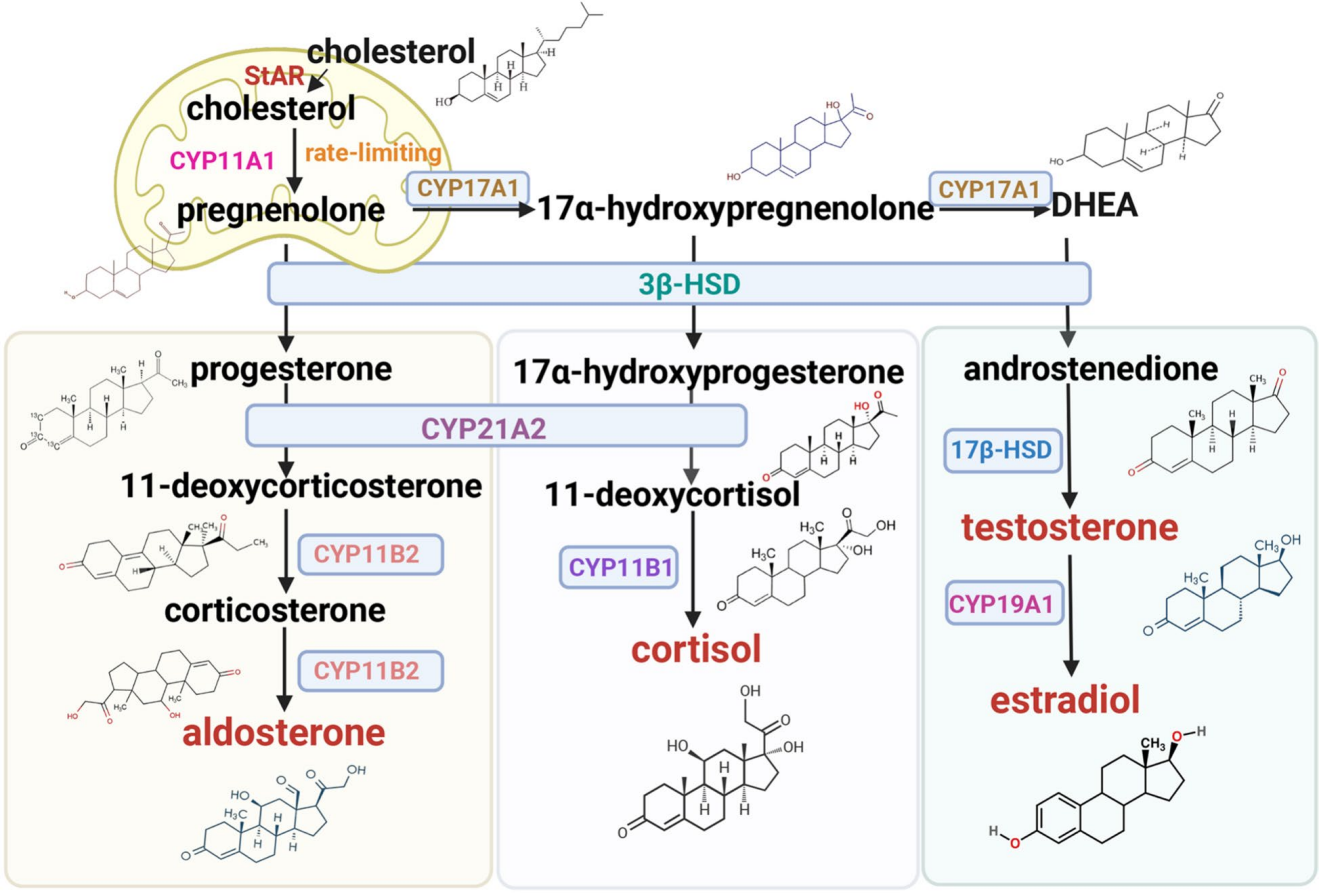
Cholesterol as a precursor to steroid hormone synthesis. The synthesis of steroid hormone synthesis begins with StAR-mediated translocation of cholesterol into the mitochondria. Within the inner mitochondrial membrane, cytochrome P450 side-chain cleavage enzyme CYP11A1 catalyzes cholesterol conversion to pregnenolone, the universal precursor for all steroid hormones. (Left) Aldosterone biosynthesis. Pregnenolone is converted to progesterone via 3β-HSD in the smooth endoplasmic reticulum. Subsequent CYP21A2-mediated C21 hydroxylation generates 11-deoxycorticosterone, which undergoes sequential modifications by CYP11B1 and aldosterone synthase CYP11B2 to yield corticosterone and ultimately aldosterone. (Middle) Cortisol production. Cortisol biosynthesis initiates with CYP17A1-mediated 17α-hydroxylation of pregnenolone to 17α-hydroxypregnenolone, followed by sequential catalysis through HSD3B2, CYP21A2, and CYP11B1 to generate 17α-hydroxyprogesterone, 11-deoxycortisol, and cortisol respectively. (Right) Sex hormone synthesis. The 17,20-lyase activity of CYP17A1 converts 17α-hydroxypregnenolone to DHEA, which diverges through 3β-HSD-1-mediated oxidation to androstenedione or 17β-HSD-1-dependent reduction to 5-androstenediol, both converging at testosterone. Subsequent enzymatic processing by aromatase (CYP19A1) or 5α-reductase yields 17β-estradiol or 5α-DHT, respectively. StAR, steroidogenic acute regulatory protein; 3β-HSD, 3β-hydroxysteroid dehydrogenase; DHEA, dehydroepiandrosterone; 5α-DHT, 5α-dihydrotestosterone

Cholesterol serves as the foundational precursor for all steroid hormones, functioning as the molecular scaffold for these critical regulators of developmental processes, metabolic homeostasis, and stress adaptation. Steroidogenesis employs two conserved enzyme systems across endocrine tissues: cytochrome P450 (CYP) oxidases and hydroxysteroid dehydrogenases (HSDs), despite gland-specific expression patterns. CYP enzymes contain conserved heme-binding domains, while HSDs lack heme but require NAD and NADP cofactors. The steroidogenic cascade initiates with the rate-limiting step: steroidogenic acute regulatory protein (StAR)-mediated translocation of cholesterol to mitochondrial inner membranes [118]. Within mitochondrial, cytochrome P450 side-chain cleavage enzyme (CYP11A1/P450scc) catalyzes cholesterol conversion to pregnenolone—the universal steroid hormone precursor (Fig. 4).

#### Aldosterone biosynthesis

As the principal Mineralocorticoid, aldosterone regulates extracellular fluid volume and electrolyte balance. Pregnenolone undergoes conversion to progesterone via 3β-hydroxysteroid dehydrogenase (3β-HSD) in the smooth endoplasmic reticulum. Subsequent CYP21A2 (P450c21)-mediated C21 hydroxylation generates 11-deoxycorticosterone, which is sequentially modified by CYP11B1 and aldosterone synthase (CYP11B2) to form corticosterone and ultimately aldosterone [119] (Fig. 4). Notably, agricultural antibiotics quinolone-1,4-dioxides and quinolone compounds suppress aldosterone via PKC/ERK-dependent CYP17A1 upregulation [120].

#### Glucocorticoids synthesis

Accounting for 95% of circulating glucocorticoids, cortisol regulates catabolic metabolism, immune responses, and stress adaptation. Its biosynthesis initiates with CYP17A1-mediated 17α-hydroxylation of pregnenolone to 17α-hydroxypregnenolone, followed by sequential catalysis through HSD3B2, CYP21A2, and CYP11B1 to generate 17α-hydroxyprogesterone, 11-deoxycortisol, and cortisol, respectively [121]. Nesfatin-1 (NESF-1) and its structural analog nesfatin-1-like peptide (NLP) exhibit dual functionality in glucocorticoid regulation. While enhancing pro-opiomelanocortin (POMC) synthesis (the ACTH precursor), these peptides directly inhibit cortisol production through ACTH-independent mechanisms. NESF-1 modulates apoptotic regulators (BAX, BCL-XL, BCL-2) and MAPK signaling cascades (ERK1/2, p38, JNK1/2) to suppress cortisol synthesis [122], while NLP reduces cortisol via AC/PKA/CREB pathway modulation in H295R cells [123].

#### Sex hormones synthesis

Sex hormones (androgens, estrogens, and progestogens) are essential regulators of reproductive development, secondary sexual characteristics, and physiological dimorphism. The 17,20-lyase activity of CYP17A1 converts 17α-hydroxypregnenolone to dehydroepiandrosterone (DHEA)—the universal precursor for gonadal steroids [124] (Fig. 4). DHEA metabolism diverges through 3β-HSD-1-mediated oxidation to androstenedione or 17β-HSD-1-dependent reduction to 5-androstenediol, both converging at testosterone. Subsequent enzymatic processing by aromatase (CYP19A1/P450aro) or 5α-reductase yields 17β-estradiol or 5α-dihydrotestosterone (5α-DHT), respectively [118].

## Diseases associated with cholesterol metabolism

**Fig. 5:**
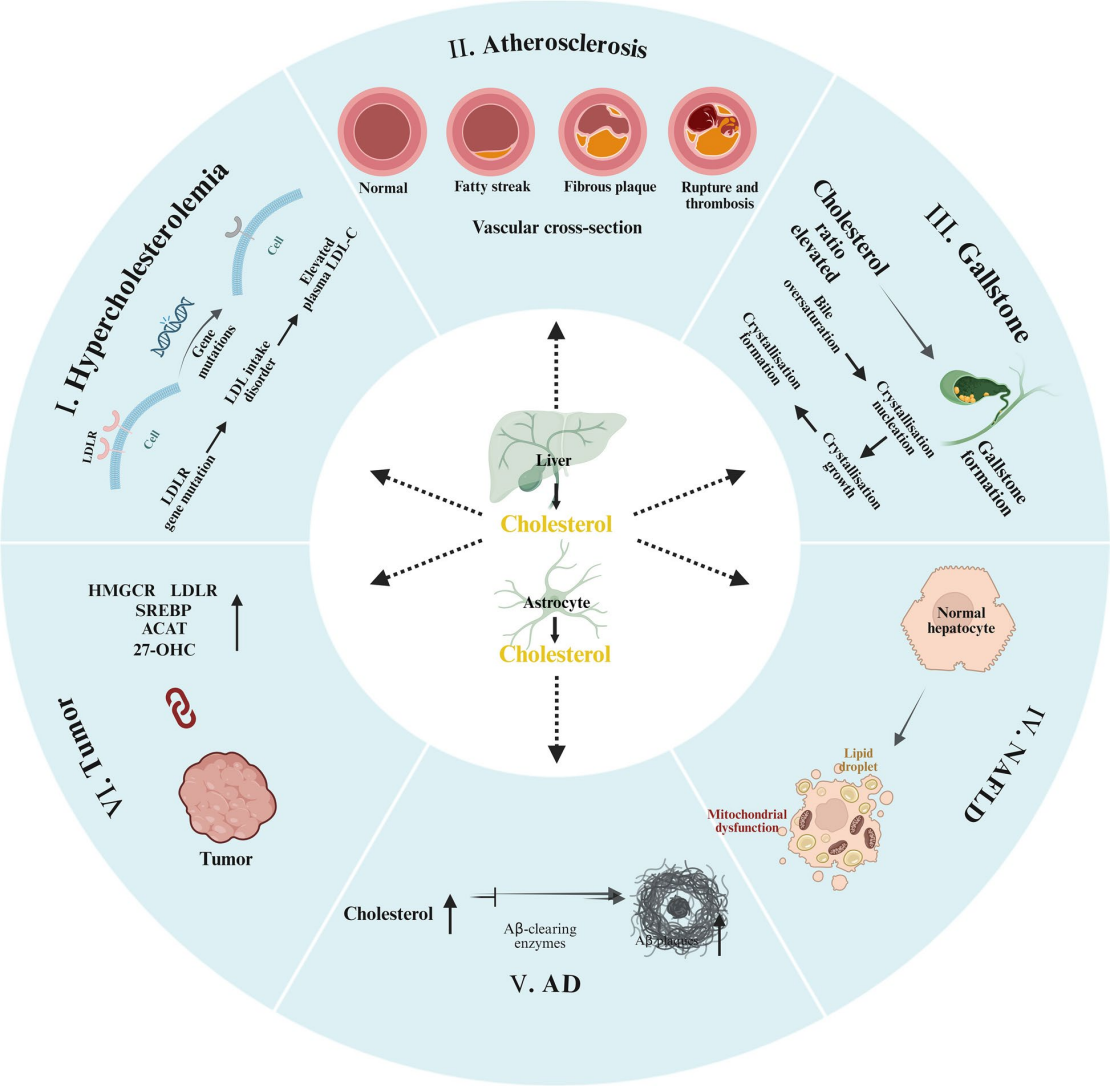
Diseases associated with cholesterol metabolism. Dysregulation in cholesterol metabolism can lead to various diseases. I. Genetic mutations in the LDLR gene reduce the quantity and functionality of LDLR, impairing cellular uptake of LDL-C and resulting in elevated plasma LDL-C levels, a condition known as FH. II. The deposition of oxidized LDL on arterial walls triggers phagocytosis by macrophages, leading to the formation of macrophage foam cell and the development of atherosclerotic plaque. III. Supersaturation of cholesterol in bile can lead to its precipitation and crystallization in the gallbladder, resulting in gallstone formation. IV. Elevated cholesterol synthesis, coupled with impaired secretion of VLDL by hepatocytes, leads to lipid accumulation, potentially resulting in NAFLD. V. Elevated cholesterol levels have been observed in individuals with Alzheimer's disease. Excess cholesterol can impede the activity of the enzyme responsible for cleaving Aβ, exacerbating its intracellular accumulation and worsening the progression. VI. Abnormalities in cholesterol metabolism are Linked to tumorigenesis. Tumor cells often exhibit increased cholesterol levels, which correlate with dysregulation of HMGCR, overexpression of LDLR, hyperactivity of ACAT, abnormal metabolism of 27-OHC, and persistent activation of the SREBP pathway. FH, familial hypercholesterolemia; Aβ, amyloid-beta; HMGCR, HMG-CoA reductase; ACAT, acyl-CoA:cholesterol acyltransferase; 27-OHC, 27-hydroxycholesterol; SREBP, sterol regulatory element-binding protein. This figure was created via BioRender

Cholesterol metabolism is characterized by a complex dynamic equilibrium. Dysregulation of cholesterol homeostasis—characterized by imbalances in synthesis, absorption, transport, or excretion—serves as a critical pathogenic driver for a spectrum of chronic and degenerative diseases (Fig. 5). These conditions span cardiovascular, hepatic, neurological, and oncological systems, reflecting the systemic impact of cholesterol dysmetabolism.

### Hypercholesterolemia

Hypercholesterolemia, a clinically significant dyslipidemia characterized by chronically elevated plasma total cholesterol (≥ 5.2 mmol/L [200 mg/dL]) and/or LDL-C levels, constitutes a major modifiable risk factor for atherosclerotic cardiovascular disease [125]. Classified per the 2024 Chinese Blood Lipid Management Guidelines and aligned with American College of Cardiology criteria, this metabolic disorder manifests in two principal etiological forms: primary (hereditary) and secondary. Primary hypercholesterolemia predominantly stems from genetic defects, most notably familial hypercholesterolemia (FH)—an autosomal dominant condition primarily driven by pathogenic variants in the LDLR gene [126]. These mutations impair LDL-C clearance, resulting in pathological LDL-C accumulation. Secondary hypercholesterolemia arises from acquired factors, including comorbidities (e.g., coronary artery disease, diabetes mellitus), pharmacological agents (e.g., diuretics, β-blockers, glucocorticoids), and lifestyle determinants. Notably, obesity amplifies risk through dysregulated lipid metabolism and hyperlipidemia, while excessive saturated fat intake, physical inactivity [127], chronic alcohol consumption, and psychosocial stress further exacerbate the disruption of cholesterol homeostasis.

Given its multifactorial nature, hypercholesterolemia can initially be managed through lifestyle interventions targeting modifiable extrinsic factors. Current strategies for intervention include [128]: 1) modifying dietary composition and structure by limiting the consumption of high-fat foods and reducing the intake of exogenous cholesterol. Research indicates that the DASH diet, which is specifically designed to prevent and manage hypertension, is effective in lowering levels of LDL-C [129]. Additionally, findings by Luiza et al. suggest that adherence to the Mediterranean diet can also ameliorate dyslipidemia in affected individuals [130]. 2) Engaging in regular physical activity to enhance metabolic processes, thereby improving abnormal Lipid metabolism; and 3) Practicing moderate alcohol consumption to mitigate overall alcohol intake. When lifestyle adjustments yield insufficient response, pharmacotherapy escalation becomes imperative. First-line agents include statins, cholesterol absorption inhibitors, inhibitors of PCSK9, bile acid sequestrants, and fibrates. These therapeutic modalities, with their distinct mechanisms and clinical applications, will be comprehensively analyzed in subsequent sections.

### Atherosclerosis

Atherosclerosis (AS) is a chronic immunoinflammatory disease of the arterial system, pathologically characterized by the accumulation of lipid-rich plaques within the vessel walls, leading to luminal stenosis and reduced vascular compliance [131]. It primarily affects large and medium-sized arteries and is driven by a variety of risk factors, among which dyslipidemia—particularly elevated LDL levels—constitutes the principal pathogenic determinant [132]. Circulating LDL particles migrate across the endothelium and undergo oxidative modification (e.g., malondialdehyde conjugation), forming pro-inflammatory oxidized LDL (oxLDL). oxLDL activates macrophage scavenger receptors (e.g., CD36, LOX-1), initiating a self-sustaining cycle of foam cell formation. Macrophages internalize oxLDL but cannot efficiently export cholesterol due to HDL dysfunction. In hypercholesterolemic conditions, the RCT system, mediated through HDL binding to hepatic SR-B1 receptors, becomes overwhelmed [133]. This promotes the development of a necrotic core and fibrous cap, which are hallmarks of vulnerable plaques. Therefore, dysregulated cholesterol metabolism—specifically, impaired lipid clearance due to elevated LDL and dysfunctional HDL—serves as the central mechanism in the pathogenesis of AS, driving the entire process from endothelial injury to plaque formation. Although hypercholesterolemia remains the cardinal modifiable risk factor, other contributors include hypertension, diabetes mellitus [134], obesity [135], and lifestyle factors such as smoking [136].

### Metabolic (dysfunction)-associated fatty liver disease (MAFLD)

MAFLD is a chronic hepatic condition characterized by hepatic lipid accumulation (steatosis) in the absence of other identifiable causes, such as genetic disorders or excessive alcohol consumption [137]. The disease spectrum ranges from simple fatty liver (FL), non-alcoholic steatohepatitis (NASH), fibrosis, and cirrhosis [138]. Its progression is driven by multiple interacting factors, including genetic predisposition, environmental influences, gut microbiota dysbiosis, oxidative stress, insulin resistance, inflammation, and dyslipidemia, which may operate in parallel or sequentially across disease stages [139–141]. Cholesterol is recognized as a principal lipotoxic agent in MAFLD pathogenesis [142], with its dysregulation playing a central role in the progression of MAFLD to NASH and fibrosis. Excessive cholesterol intake and accumulation promote disease progression, and cholesterol-induced inflammatory responses identified as a critical contributor to these conditions [143].

Management of MAFLD includes both non-pharmacological and pharmacological strategies, tailored to individual risk profiles. Dietary modifications, such as Mediterranean diet [143] and the ketogenic diet [144], are effective therapeutic options. Regular physical exercise also demonstrates beneficial effects in MAFLD treatment [145]. The gut-liver axis play an important role, as gut health significantly influences hepatic function, making gut microbiota a potential therapeutic target [146]. For example, probiotics may modulate gut microbiota composition and exert beneficial effects on MAFLD [147], and certain flora characteristics might serve as diagnostic or prognostic biomarkers [147]. However, a 2021 study conducted by Nor et al. suggested that six months of probiotic supplementation did not yield significant clinical improvement, indicating that probiotics may be more suitable as adjunctive rather than stand-alone therapy [148]. The first pharmacological treatment for NASH is Rezdiffra [149], an oral THR-β agonist that selectively activates hepatic thyroid hormone receptor β. It modulates lipid metabolism, promotes energy expenditure, and reduces liver fat and inflammation. Other pharmacological approaches target underlying pathogenesis, including lipid-lowering agents (e.g., statins), PPARα agonists (e.g., obeticholic acid), and FXR agonists (e.g., cilofexor and elafibranor).

### Gallstones

Gallstones are solid crystalline deposits that form in the gallbladder or biliary tract, representing a common gastrointestinal disorder. They are mainly classified into two types: cholesterol stones and pigment stones. The pathogenesis of gallstone disease is multifactorial, involving a complex interaction of genetic predispositions, lifestyle factors, and other influences. The predominant mechanism underlying gallstone formation is cholesterol supersaturation in bile, which drives the development of cholesterol stones. Under physiological conditions, cholesterol is solubilized within micelles and vesicles formed by bile acids and lecithin. However, when cholesterol levels exceed the solubilizing capacity of these compounds, it leads to supersaturation and subsequent crystallization. Notably, recent studies suggest that cholesterol supersaturation in gallstone patients may be primarily due to a deficiency in bile acids rather than excessive cholesterol production [150].

Treatment strategies for gallstones include pharmacological interventions and surgical procedures. Ursodeoxycholic acid (UDCA) is a standard medication for cholesterol gallstones, as it promotes stone dissolution by increasing bile acid secretion and suppressing hepatic cholesterol synthesis, thereby reducing cholesterol saturation in bile. Statins have also emerged as a potential treatment due to their ability to modulate cholesterol metabolism and decrease gallstone formation. According to Georgescu et al. [151], statins may additionally function through the modulation of intestinal microbiota. Traditional Chinese medicine further contributes to gallstone management. For example, Huang et al. [152] reported that Ganoderma lucidum polysaccharides alleviate cholesterol gallstone formation via FXR-dependent regulation of cholesterol and bile acid metabolism. Other herbal formulations [153], such as Shugan Lidan Xiaoshi Granules (SLXG), have also demonstrated therapeutic potential by targeting genes including HMGCR, SOAT2, and UGT1A1, thereby modulating cholesterol homeostasis [154]. Additionally, growing evidence implicates the gut microbiota in the prevention and treatment of cholesterol gallstones. For instance, Wang et al. [155] showed that lactobacilli supplementation reduced the incidence and severity of high-fat diet-induced gallstones, pointing to a promising new therapeutic direction.

### Neurodegenerative diseases

**Fig. 6:**
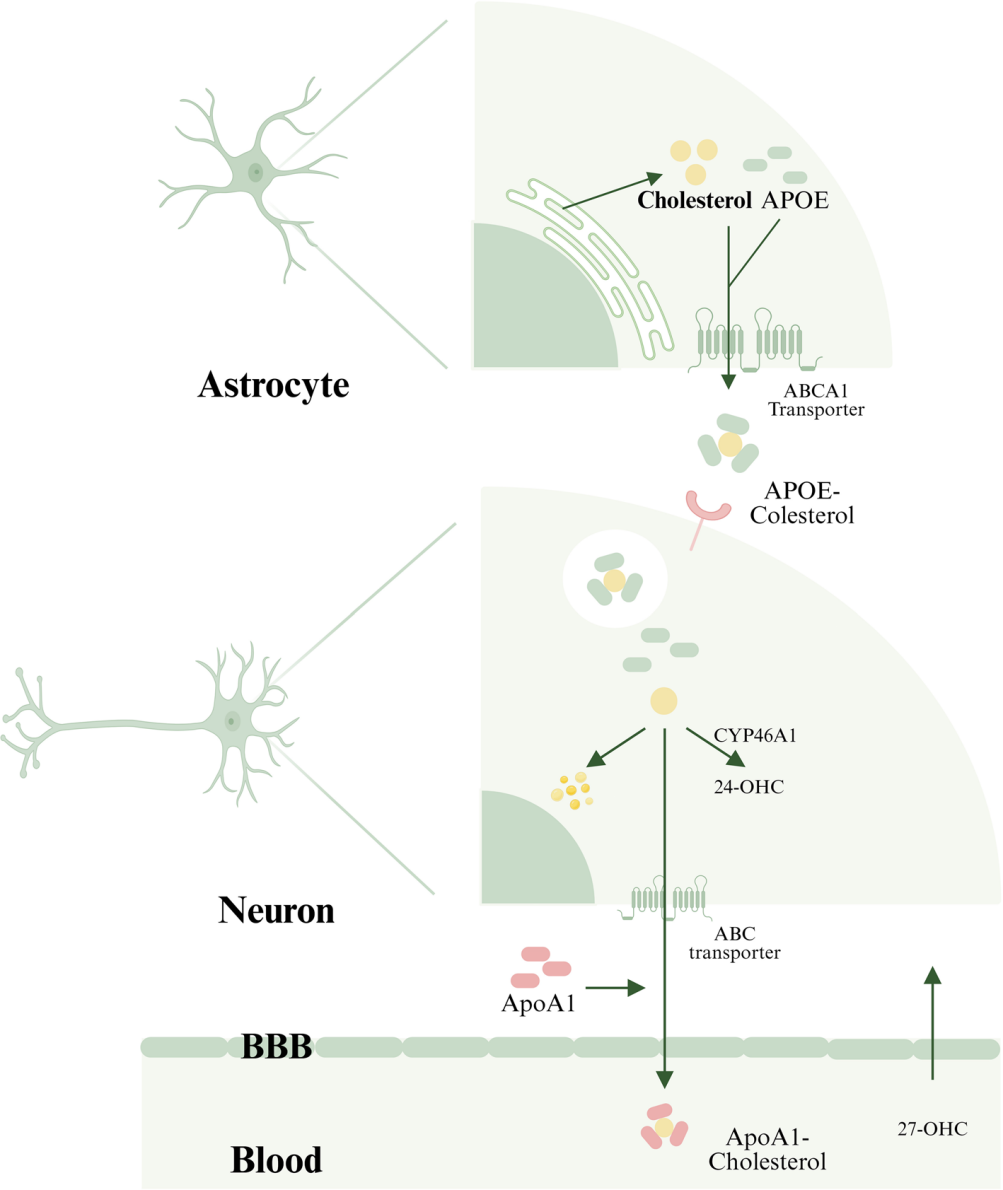
Potential impact of cholesterol in Alzheimer's disease pathology. Cholesterol metabolism in the brain follows a complex sequence of events. Initially, cholesterol is synthesized within the endoplasmic reticulum of astrocytes. It then associates with APOE to form APOE-cholesterol granules. These granules are secreted into the extracellular fluid, a process facilitated by ABC transporter proteins. Subsequently, cholesterol is internalized by neurons that express LDLRs. Within neuronal cells, cholesterol undergoes three distinct metabolic pathways. A portion of cholesterol is converted into lipid droplets for intracellular storage, while another fraction is released from the cell and combines with ApoA1 for direct excretion. The majority of cholesterol is metabolized by CYP46A1, resulting in the production of 24-OHC. This metabolite can cross the blood–brain barrier and entering the plasma, where it facilitates the influx of 27-OHC into the brain. ABC, ATP-binding cassette; 24-OHC, 24-hydroxycholesterol; 27-OHC, 27-hydroxycholesterol; CYP46A1, cholesterol 24-hydroxylase. This figure was created via BioRender

The Human brain contains the highest cholesterol concentration among all organs, accounting for approximately 25% of total body cholesterol [156]. This tightly regulated pool is essential for maintaining neuronal architecture, synaptic plasticity, and neurotransmission, with emerging evidence linking dysregulated cholesterol metabolism to neurodegenerative diseases [157, 158]. Segregated from systemic circulation by the blood–brain barrier (BBB), cerebral cholesterol operates as an autonomous metabolic system. Astrocytes are the primary source of brain cholesterol, producing it through de novo synthesis and delivering it to neurons via ApoE-mediated transport. Excess cholesterol undergoes three regulatory fates (Fig. 6): (1) storage as cytoplasmic lipid droplets, (2) efflux mediated by ABC transporters via ApoA1-bound particles, or (3) enzymatic conversion to oxysterols (24-hydroxycholesterol [24-OHC] and 27-OHC) for elimination across the BBB [159].

Alzheimer's disease (AD), a prototypical neurodegenerative disorder, characterizes by amyloid-β (Aβ) plaque deposition and neurofibrillary tangles. Increased cholesterol levels in neuronal membranes correlates with AD progression [160, 161]. Hypercholesterolemia promotes Aβ production by facilitating amyloid precursor protein (APP) processing within cholesterol-rich lipid raft via β- and γ-secretases. The ApoE4 allele (ε4), the strongest genetic risk factor for late-onset AD, impairs cholesterol transport and Aβ clearance. Notably, oxysterol dynamics demonstrate opposing roles: 27-OHC exacerbates Aβ production and AD progression, whereas 24-OHC promotes α-secretase activity to reduce Aβ burden, exerting neuroprotective effects [159].

The limited clinical success of current Aβ-targeted therapies has shifted focus toward cholesterol metabolism interventions. Statins, traditionally used for dyslipidemia, show potential in AD Management. A meta-analysis of 55 observational studies indicated that statin therapy is associated with a 14% reduction in dementia risk and an 18% decrease in AD incidence [162]. These benefits may arise from inhibited cholesterol synthesis and reduced Aβ aggregation, particularly with BBB-penetrating lipophilic statins such as simvastatin [163, 164]. Emerging evidence highlights the therapeutic relevance of CYP46A1, a neuron-specific enzyme that converts cholesterol to 24-OHC. Overexpression of CYP46A1 improves cognition in mouse models [165], and its pharmacological modulation shows promise: for example, KaiXinSan upregulates CYP46A1 to alleviate 27-OHC-induced cognitive deficits [166], while the HIV drug efavirenz acts as a concentration-dependent modulator of CYP46A1 [167]. Novel efavirenz analogs designed specifically to target CYP46A1 activity offer further potential for AD treatment [168]. Together, these findings establish cholesterol metabolic regulation as a promising therapeutic frontier in AD pathogenesis [158].

### Tumors

Emerging evidence identifies dysregulated cholesterol metabolism as a critical metabolic hallmark of cancer. Compared to normal cells, malignant cells exhibit profound disruptions in cholesterol homeostasis [169], characterized by four key alterations: (1) hyperactivation of de novo cholesterol biosynthesis, (2) enhanced LDLR-mediated uptake, (3) impaired cholesterol efflux, and (4) pathological accumulation of cholesterol derivatives [170, 171]. Comparative studies indicate that tumor tissues harbor significantly higher cholesterol levels than adjacent normal tissues, which correlates with increased aggressiveness and metastatic potential [3]. This is underpinned by marked upregulation of genes involved in cholesterol biosynthesis, such as *SREBP2*, *HMGCR*, *SQS*, *OSC*, and *SQLE* [172], along with elevated LDLR expression and enhanced cholesterol esterification [173].

Current anticancer strategies targeting cholesterol metabolism primarily focus on inhibiting biosynthesis, transport, and esterification. Statins, classical HMGCR inhibitors, exhibit broad-spectrum anticancer activity in prostate, gastric, esophageal, and breast cancers. They act by depleting cellular cholesterol levels and suppressing isoprenoid synthesis (e.g., geranylgeranyl pyrophosphate (GGPP) and FPP), thereby disrupting GTPase function in cancer cells [170, 173, 174]. However, long-term statin use (> 4 years) has been associated with an increased risk of several cancers, including those of the colon, bladder, and lung [175]. Beyond cholesterol-lowering effects, statins exert anticancer actions through multiple mechanisms: inducing apoptosis via FOXO3a activation and autophagy inhibition in oral and colon cancers [176], triggering ferroptosis via mevalonate pathway disruption [177, 178], promoting pyroptosis [179], and modulating the tumor microenvironment [180]. Clinically, stains show synergistic effects when combined with conventional therapies, improving outcomes in esophageal squamous cell carcinoma patients receiving chemoradiotherapy [181] and overcoming drug resistance in recurrent small-cell lung cancer [182]. To reduce side effects such as myopathy, nano-formulated statin with improved tumor targeting are under development [183].

The transcription factor SREBP2 is a critical regulator of cholesterol synthesis. Its activation by phosphate-dependent prolyl isomerase (PIN1)-mediated isomerization promotes oncogenic cholesterol accumulation, and PIN1 silencing reduces cellular cholesterol levels, showing therapeutic potential in bladder cancer [184]. Inhibiting cholesterol transport through blocking lysosomal cholesterol release [185, 186] or targeting NPC1with agents like itraconazole [187] also effectively suppresses tumor growth. Additionally, cholesterol esterification through ACAT/SOAT1 represents a vulnerable node, with inhibitors such as avasimibe exerting potent antitumor effects in melanoma models [188]. Clinical evidence shows pathological cholesteryl esters accumulation in pancreatic [189], colorectal [190], and metastatic prostate cancer [191], where ACAT1 inhibition substantially reduces progression and metastasis.

While targeting cholesterol metabolism holds broad anticancer potential, growing evidence suggests the need for cancer type-specific therapeutic approaches. Current strategies include inhibiting synthesis, disrupting transport, and blocking esterification, each requiring tailored application based on tumor metabolic dependencies. Future directions involve developing precision therapies that combine metabolic targeting with conventional treatments and advanced delivery systems such as nanotechnology.

## Cholesterol-lowering therapies: from established agents to precision-targeted strategies

Cholesterol metabolism dysregulation lies at the heart of cardiovascular diseases, neurodegenerative diseases, and metabolic syndrome. After half a century of exploration, cholesterol management strategies have evolved from broad-spectrum inhibition of synthesis to precise regulation of metabolic networks (Table 1).

**Table 1:** Cholesterol-lowering therapies

Drug Class	Specific drugs	Mechanism	Side effects	Progress
Existing drugs				
Bile Acid Sequestrants	Cholestyramine	Bind bile acids in the intestine, reducing reabsorption and increasing cholesterol conversion	Gastrointestinal issues (e.g., constipation, bloating)	Established therapy; limited by tolerability
	Colesevelam	Same as above, with enhanced bile acid binding	Gastrointestinal issues; increases triglycerides	Newer agent with fewer side effects
Niacin	Niacin	Inhibits lipolysis, reducing VLDL/LDL, increasing HDL	Flushing, gastrointestinal upset, increased glucose	Declining use due to side effects and debated benefits
Statins	Atorvastatin Rosuvastatin Simvastatin Pravastatin	Inhibit HMGCR, reducing cholesterol synthesis	Muscle pain, liver enzyme elevation, diabetes risk	Widely used; ongoing combination studies
Cholesterol Absorption Inhibitors	Ezetimibe	Inhibits intestinal cholesterol absorption, increasing LDLR expression	Well-tolerated; few side effects	Proven benefit with statins (IMPROVE-IT trial)
PCSK9 monoclonal antibodies	Evolocumab Alirocumab	Bind PCSK9, preventing LDL receptor degradation	Injection site reactions; well-tolerated	Approved for high-risk patients; long-term studies ongoing
PCSK9 siRNA	Inclisiran	siRNA silences PCSK9 expression	Injection site reactions; well-tolerated	Approved in 2021; biannual dosing studied
ATP citrate lyase inhibitors	Bempedoic acid	Inhibits ATP citrate lyase, reducing cholesterol synthesis	No significant muscle side effects	Approved in 2020 for statin-intolerant patients
Fibrates	Fenofibrate	Activate PPARα, reducing triglycerides, increasing HDL	Gastrointestinal issues, myopathy with statins	Used for hypertriglyceridemia; limited CVD benefit
	Bezafibrate	Same as above, with broader PPAR activity	Same as above	Same as above
MTP inhibitors	Lomitapide	Inhibits MTP, reducing VLDL and chylomicron assembly	Gastrointestinal issues, hepatic steatosis	Approved in 2012 for HoFH; limited use
ANGPTL3 Inhibitors	Evinacumab	Inhibits ANGPTL3, reducing triglycerides and LDL	Well-tolerated	Approved for HoFH; broader use explored
Future drugs				
CETP inhibitors	Obicetrapib	Inhibits CETP, increasing HDL-C, reducing LDL-C and Lp(a)	Well-tolerated; no significant issues	Phase 3 TANDEM trial positive (NCT06005597); awaiting FDA approval
Lp(a) therapies	Olpasiran	Lp(a) siRNA	Well-tolerated; minor injection site reactions	Phase 3 OCEAN(a) trial (NCT05581303); results expected 2027
	Pelacarsen	Antisense oligonucleotide targeting Lp(a)	Well-tolerated	Phase 3 HORIZON trial (NCT04023552); results expected 2026
Gene editing	VERVE-101	CRISPR edits PCSK9 gene, permanently reducing PCSK9	Unknown; early-stage risks unclear	Phase 1 trials (NCT05398029); promising initial data
PCSK9 oral Inhibitors	MK-0616	Oral small molecule inhibits PCSK9	Well-tolerated; limited data	Phase 2 trials (NCT06008756); Phase 3 data expected 2026
ANGPTL3 inhibitors	Zodasiran	ANGPTL3 siRNA	Well-tolerated	Phase 2 trials (NCT04832971); further data expected
	Vupanorsen	ANGPTL3 antisense oligonucleotide	Well-tolerated	Preclinical to early clinical stages
ApoC3 inhibitors	Volanesorsen	ApoC3 antisense oligonucleotide	Injection site reactions, thrombocytopenia	Approved for FCS; broader use studied
	AKCEA-ApoC3-LRx	ApoC3 siRNA	Well-tolerated	Phase 2/3 trials (NCT05130450); data expected 2026
LXR agonists	Various	Promote RCT, reduce inflammation	Potential metabolic side effects	Preclinical; selectivity challenges
Other emerging therapies	SR-BI modulators, LCAT activators	Various mechanisms to lower lipids	Limited data	Early research; long-term potential unclear

Data sources: clinical trials website (https://clinicaltrials.gov/)

Abbreviations: ANGPTL3, angiopoietin-like 3; APOC3, Apolipoprotein C3; ATP, adenosine triphosphate; CETP, cholesterol ester transfer protein; CVD, cardiovascular disease; FCS, familial chylomicronemia syndrome; HDL, high-density Lipoprotein; HMGCR, 3-hydroxy-3-methylglutaryl-CoA reductase; HoFH, homozygous familial hypercholesterolemia; LCAT, lecithin-cholesterol acyltransferase; LDL, low-density lipoprotein; LDLR, low-density lipoprotein receptor; Lp(a), lipoprotein(a); LXR, liver X receptor; MTP, microsomal triglyceride transfer protein; PCSK9, proprotein convertase subtilisin/kexin type 9; PPARα, peroxisome proliferator-activated receptor; RCT, reverse cholesterol transport; siRNA, small interfering RNA; SR-BI, scavenger receptor class B type I; VLDL, very-low-density lipoprotein

### Existing medications

The classic therapy, exemplified by statins, has revolutionized cardiovascular disease prevention by inhibiting HMGCR. Despite their efficacy, statin intolerance and residual cardiovascular risk persist, particularly in genetic disorders like HoFH, propelling the development of next-generation therapies. A significant milestone in this evolution is the advent of PCSK9 inhibitors. Monoclonal antibodies, such as evolocumab and alirocumab, achieve substantial LDL-C reduction by blocking the PCSK9-LDLR interaction [192]. The paradigm further shifted with inclisiran, an siRNA therapy that enables biannual dosing through GalNAc-mediated hepatocyte targeting. This exemplifies the transition from chronic treatment to durable gene-silencing strategies. In addition, non-statin agents like ezetimibe and bile acid sequestrants have expanded the therapeutic arsenal. They address intestinal cholesterol absorption and bile acid recirculation. For refractory cases like HoFH, ANGPTL3 inhibitors (such as evinacumab) and MTP inhibitors (such as lomitapide) open up survival pathways for patients who previously had "no drugs available" through LDLR-independent pathways.

While these interventions effectively reduce circulating LDL-C, their fundamental approach of suppressing endogenous cholesterol bioavailability warrants critical scrutiny. Given cholesterol’s indispensable role in membrane integrity, neurosteroid synthesis, and bile acid production, the long-term consequences of systemic cholesterol depletion remain inadequately characterized. Emerging evidence of statin-associated adverse effects (e.g., increased diabetes risk, myopathy, and paradoxical cancer incidence with prolonged use) and the "niacin paradox" (LDL-C reduction without CVD benefit) underscore potential limitations of this strategy [193].

#### Statins serving as HMGCR inhibitors

The discovery of statins Marks a landmark achievement in the field of cholesterol Management. In 1973, Akira Endo's groundbreaking screening of 3,800 fungal strains led to the discovery of mevastatin (compactin), the first identified HMGCR inhibitor [194]. This pivotal finding paved the way for Merck Pharmaceuticals' Subsequent development of lovastatin in 1987, which became the first clinically approved statin [195]. Through continuous molecular optimization, successive generations of statins achieved enhanced potency and specificity—notably simvastatin (Zocor®), atorvastatin (Lipitor®), and rosuvastatin (Crestor®)—demonstrating LDL-C reduction efficacy ranging from 25 to 60% across clinical trials. As the most prescribed cholesterol-lowering agents worldwide, statins remain the therapeutic cornerstone for hypercholesterolemia management.

Mechanistically, statins exert their effects by inhibiting HMGCR activity [196], thereby reducing cholesterol synthesis and activating SREBP2 in the endoplasmic reticulum. Upon binding to the sterol regulatory elements (SRE) in the LDLR promoter, SREBP2 enhances LDLR synthesis [197], leading to increased uptake of circulating LDL-C. This finely tuned feedback mechanism balances intracellular cholesterol levels while reducing circulating LDL-C concentrations. Notably, statins confer persistent cardiovascular protection through pleiotropic effects, including plaque stabilization and endothelial function improvement, which contribute to residual risk reduction even after treatment cessation. However, dose-dependent adverse effects, such as statin-associated muscle symptoms (SAMS), asymptomatic hepatic transaminase elevation, plaque calcification, and rare rhabdomyolysis, necessitate careful clinical monitoring [7, 198].

#### PCSK9 inhibitors

PCSK9 inhibition has emerged as a promising strategy for managing LDL-C, delivering substantially greater efficacy than conventional statin therapy, particularly for patients with FH or statin intolerance [199]. Although adverse events such as influenza‐like illness, nasopharyngitis, myalgia, and injection‐site reactions (ISRs) have been reported, these are typically transient and self-resolving [200]. Importantly, PCSK9 inhibitors exhibit a favorable safety profile with a markedly lower risk of myopathy compared to statin-associated muscle complications. Additionally, when combined with statins, they enable further reductions in LDL-C levels [201].

Two landmark fully human monoclonal antibodies (mAbs) targeting PCSK9, evolocumab (Repatha®, Amgen) and alirocumab (Praluent®, Regeneron/Sanofi), have received global approved for treating hypercholesterolemia. By binding to the LDLR-interacting domain of PCSK9, they effectively prevent PCSK9-LDLR interaction. In combination with statin therapy, these antibodies can reduce LDL-C by up to 72% [192]. Several other PCSK9-targeting mAbs have been developed, including bococizumab (Pfizer), frovocimab/LY3015014 (Eli Lilly), 1B20 (Merck), JS002 (Junshi Bio), recaticimab, as well as the China-approved agents tafolecimab/IBI306 (2023) [202] and ebronucimab/AK102 (2024) [203] (Dawnrays Biotechnology Capital). Among these, bococizumab and frovocimab have shown LDL-C-lowering effects comparable to alirocumab and evolocumab. Notably, frovocimab employs a distinctive dual-action mechanism, its binding not only blocks PCSK9-LDLR interaction but also facilitates furin-mediated proteolytic inactivation of PCSK9, thereby reducing systemic PCSK9 accumulation and enabling extended therapeutic effects [204].

A paradigm shift occurred with the 2021 FDA approval of inclisiran (Leqvio®, Alnylam/Novartis), the first siRNA-based PCSK9 inhibitor. This agent utilizes a triantennary GalNAc conjugate for hepatocyte-specific delivery, forming a subcutaneous depot that enables sustained intracellular release and prolonged PCSK9 inhibition [205]. Its innovative pharmacokinetics allow for biannual dosing – a significant advantage over the biweekly or monthly injections required for mAbs (13–26 injections annually) [206]. Clinical data confirm durable efficacy, with inclisiran Maintaining a 44.3% reduction in LDL-C from day 90 to 540 after injection [207]. Supported by consistent efficacy and safety over 5-year follow-up along with its infrequent dosing, inclisiran offers a more convenient treatment option [208, 209]. The ongoing ORION-4 clinical trial (NCT03705234) is evaluating Inclisiran’s effects in 15,000 patients with atherosclerotic cardiovascular disease (ASCVD) [210], with the potential to redifine long-term ASCVD management strategies.

#### Ezetimibe targeting NPC1L1 receptor

Originally developed by Schering-Plough Pharmaceuticals, ezetimibe represents the first-in-class cholesterol absorption inhibitor that specifically targets the NPC1L1 receptor [211]. Mechanistically, this therapeutic agent reduces intestinal cholesterol absorption through selective inhibition of the NPC1L1 receptor-mediated cholesterol transport system in the brush border membrane of enterocytes. By competitively binding to the extracellular domain of NPC1L1, ezetimibe effectively blocks the internalization process of cholesterol and phytosterols from intestinal micelles, thereby preventing their subsequent incorporation into chylomicrons for hepatic delivery [212, 213]. This pharmacological action creates a dual therapeutic effect: (1) direct reduction of dietary cholesterol absorption, and (2) indirect enhancement of circulating LDL clearance. The decreased hepatic cholesterol influx triggers compensatory upregulation of LDLR on hepatocytes, significantly improving the clearance of circulating LDL particles from the bloodstream [214]. Notably, ezetimibe's therapeutic activity is predominantly localized within the enterohepatic circulation system, resulting in minimal systemic exposure (bioavailability < 5%) and consequently low risks of pharmacokinetic drug-drug interactions [215]. Clinically, ezetimibe demonstrates synergistic effects when combined with statin therapy, producing an incremental 15–20% reduction in LDL-C levels beyond statin monotherapy. This combination strategy has been validated in multiple randomized controlled trials to significantly improve cardiovascular outcomes in high-risk populations [216]. The safety profile of ezetimibe remains favorable, with extensive clinical trials documenting no significant increase in serious adverse effects compared to placebo, making it particularly suitable for long-term lipid management.

#### Cholestyramine and colestipol: bile acid sequestrants (BASs)

BASs are cationic polymeric gels that lower plasma LDL-C by interrupting enterohepatic bile-acid cycling [217]. In the intestinal lumen these positively charged macromolecules bind conjugated bile acids via ionic interactions, forming insoluble complexes that are excreted in the stool and deplete the total bile-acid pool by ≥ 40%. This triggers a hepatic compensatory response, upregulating cholesterol 7α-hydroxylase (CYP7A1) and thereby accelerating the conversion of cholesterol into new bile acids. The increased demand for cholesterol subsequently prompts the upregulation of hepatic LDLRs, enhancing LDL clearance and reducing circulating LDL-C [218]. BASs tend to increase the concentration of bile acids in the gastrointestinal tract, which may lead to gastrointestinal side effects such as constipation, diarrhea, bloating, nausea, abdominal pain, and weakness [219]. Because BASs can also impair absorption of fat-soluble vitamins and lipophilic drugs (e.g., statins), they should be taken at least four hours before such agents [220]. Despite these tolerability challenges, BASs remain a safe, non-systemic option for cholesterol reduction. Currently, the only BAS that have received approval for clinical use are colesevelam hydrochloride and colestilan [221]. Colesevelam with its engineered polyallylamine backbone enriched in hydrophobic domains offers the highest bile-acid binding capacity and the lowest rate of adverse effects [222], although it can still provoke the class-typical gastrointestinal symptoms and, notably, may raise triglyceride levels [223].

#### Niacin

Niacin, also known as vitamin B3 or nicotinic acid, is an essential micronutrient required for NAD synthesis [224]. Historically, niacin has been a first-line therapy for dyslipidemia [225]. By inhibiting adipose-tissue lipolysis, it cuts the Supply of free fatty acids needed for hepatic triglyceride synthesis, thereby lowering VLDL production and, secondarily, circulating LDL-C; simultaneously, it remains the most potent agent for raising HDL-C and can reduce triglycerides by 20–35% [226]. These effects made niacin a valuable adjunct for familial hyperlipidemia and other severe hypercholesterolemia. In the statin era, however, large outcome trials show that adding niacin to intensive statin therapy, despite further improving the lipid profile, confers no additional cardiovascular risk reduction [227] and may even increase overall mortality [228]—an observation termed the “niacin paradox.” This suggests that niacin’s influence on cardiovascular events involves pathways that are either independent of, or inadequately captured by, its traditional cholesterol-modulating mechanisms [229].

#### Bempedoic acid

Bempedoic acid is an oral, once-daily prodrug that is selectively activated in hepatocytes by very-long-chain acyl-CoA synthetase 1 (ACSVL1, encoded by the *SLC27A2*). Upon activation, bempedoic acid is converted into bempedoic acid-CoA, which serves as a potent and selective inhibitor of ATP-citrate lyase (ACLY), the enzyme that catalyzes the committed step in cholesterol and fatty acid biosynthesis [230]. By curtailing hepatic cholesterol biosynthesis, bempedoic acid triggers SREBP2-mediated up-upregulation of LDLR, accelerating LDL-C clearance and lowering plasma LDL-C levels. Because ACSVL1 is absent from skeletal muscle, bempedoic acid avoids the myalgic side-effects common to statins, making it an FDA-approved (February 2020) stand-alone or add-on therapy—marketed as Nexletol—for patients who are statin-intolerant [231].

#### Treatment of homozygous familial hypercholesterolemia (HoFH)

Familial hypercholesterolemia (FH) is primarily caused by pathogenic variants in the *LDLR* gene or its associated proteins. Homozygous FH (HoFH) represents the most severe form of the disorder, resulting from biallelic mutations in *LDLR* or related genes [232]. Given the central role of LDLR in LDL metabolism, most lipid-lowering therapies—including statins, ezetimibe, PCSK9 inhibitors, and BASs—function by upregulating LDLR expression on the cell surface. However, most HoFH patients exhibit poor or no response to LDLR-targeting therapies [233]. Historically, liver transplantation, ileal bypass surgery, and portacaval shunt procedures were used in early treatment of HoFH. However, the latter two approaches have largely been abandoned due to efficacy and safety concerns [232].

Currently, LDL apheresis, MTP inhibitors, and ANGPTL3 inhibitors are recognized as LDLR-independent therapeutic options for HoFH [234]. LDL apheresis, initially performed using plasma exchange, has now largely been replaced by more selective LDL removal techniques [235]. Lomitapide, an MTP inhibitor, lowers lipid levels by reducing the production of chylomicrons and VLDL and is approved exclusively for HoFH treatment [236]. Evinacumab, a monoclonal antibody targeting ANGPTL3, has been approved for HoFH therapy and can reduce LDL-C levels by approximately 50% regardless of residual LDLR function [237]. Beyond HoFH, evinacumab has demonstrated efficacy in refractory hypercholesterolemia and severe hypertriglyceridemia, though it is not indicated for patients with persistent chylomicronemia due to impaired LPL bioavailability [238]. Effective LDL-C control in HoFH typically requires a combination of therapies with distinct mechanisms of action. In the HICC cohort, only 2.6% of patients achieved LDL-C targets with monotherapy, whereas 53.3% of those receiving five different treatments met target LDL-C levels [239].

#### Fibrates (fibric acid)

Fibrates lower plasma triglycerides and raise HDL-C by selectively activating the nuclear receptor PPARα. Upon activation, PPARα forms a heterodimer with RXR and transcriptionally activates genes governing fatty acid uptake, β-oxidation, and lipoprotein metabolism [240–242]. PPARα simultaneously upregulates lipoprotein lipase (LPL) expression and represses its inhibitor ApoC3, accelerating the hydrolysis of triglyceride-rich VLDL and chylomicrons. Fibrates also increase HDL-C levels by stimulating the synthesis of ApoA1 and ApoA2, the principal scaffolding proteins of HDL. Beyond lipid modulation, PPAR-α also dampens vascular inflammation by blocking NF-κB–mediated transcription of TNF-α, IL-1β and other pro-inflammatory cytokines, an effect that slows atherogenesis independent of lipid changes [240].

Although PPARα remains the canonical target, individual fibrate drugs differ in receptor breadth. Bezafibrate, a weak pan-PPAR ligand, activates PPARAα, PPARγ, and PPARβ/δ to varying degrees, contributing to its multifaceted effects [243]. PPAR-γ activation further enhances anti-inflammatory responses by inhibiting TNF-α and IL-1β, while PPAR-β/δ modulates lipid metabolism and bile acid homeostasis, though its role remains less defined. Dual PPAR agonists, such as saroglitazar (PPARα/γ) and elafibranor (PPARα/δ), further expand the therapeutic scope of fibrates. Notably, elafibranor and the selective PPAR-δ agonist seladelpar have recently received FDA approval as second-line therapies for primary biliary cholangitis, where their combined lipid-regulatory and cholestatic anti-inflammatory actions improve clinical outcomes [244].

#### Lomitapide

Lomitapide (Juxtapid) is a selective MTP inhibitor, approved in December 2012 for the treatment of HoFH in adults [245]. It effectively reduces LDL-C levels through a mechanism independent of LDLR functionality, addressing a critical therapeutic need in patients with severe dyslipidemia unresponsive to conventional therapies [246].

MTP, an intracellular protein located in the endoplasmic reticulum of hepatocytes and enterocytes, plays a pivotal role in lipid metabolism by facilitating the transfer of triglycerides to newly synthesized ApoB [247]. This process is essential for the assembly and secretion of VLDL in the liver and chylomicrons in the intestine. Lomitapide inhibits MTP activity, disrupting the coupling of triglycerides with ApoB-100 in the liver and ApoB-48 in the intestine. Consequently, it reduces the production and secretion of VLDL and chylomicrons, leading to significant reductions in plasma levels of LDL-C, ApoB, triglycerides, non-HDL-C, and lipoprotein(a) [Lp(a)]. The lipid-lowering effect of lomitapide is particularly valuable in HoFH. By bypassing the LDL receptor pathway, lomitapide provides a unique therapeutic strategy for managing severe hypercholesterolemia in this population [248].

### Novel treatment strategies

Beyond small molecules, CRISPR-based therapies like VERVE-101 promise single-dose PCSK9 inactivation, and GalNAc-conjugated antisense oligonucleotides effectively suppress ApoC3, redefining precision in triglyceride management. The therapeutic frontier now integrates multi-omics insights, targeting regulators like Lp(a) via siRNA (olpasiran), modulating LXRs to enhance reverse cholesterol transport, and exploring CETP inhibitors with refined HDL remodeling capabilities. Concurrently, natural products (berberine, red yeast rice) and microbiome-modulating supplements complement pharmacological strategies, underscoring the importance of holistic approaches.

#### Non-antibody PCSK9 inhibitors

Verve Therapeutics has pioneered a groundbreaking approach with VERVE-101, a first-in-class CRISPR/Cas9-based gene-editing therapy currently undergoing Phase I clinical evaluation (NCT05398029). This single-dose therapeutic intervention is designed to achieve permanent PCSK9 gene silencing through precise A-to-G base editing at a specific hepatic locus, potentially offering durable lipid-lowering effects [249]. Building on the clinical success of monoclonal antibodies, innovative vaccine strategies have emerged. The AT04A vaccine employs the N-terminal domain of PCSK9 as its antigenic component. Preclinical studies in murine models demonstrated sustained anti-PCSK9 antibody production, resulting in significant reductions in plasma lipid levels, mitigation of systemic and vascular inflammation, and attenuation of aortic lesions [250].

Additionally, small peptides have been investigated as PCSK9 inhibitors. PEP2-8, a peptide targeting the EGF-A binding site of PCSK9, effectively preserves LDLR expression in vitro [251]. However, its clinical translation is limited by poor oral bioavailability, necessitating intravenous administration for LDL-C reduction in vivo. MK-0616 (Merck) represents the most advanced oral candidate, this macrocyclic peptide features a tricyclic architecture that enhances both chemical stability and binding affinity while achieving relevant oral absorption [252]. Promising Phase I and II clinical trial results position it as a potential game-changer in dyslipidemia management [253]. Several non-injectable PCSK9 inhibitors capable of significantly reducing LDL-C levels are currently in development. NN6434 (NNC0385-0434) progressed to Phase II trials before development was halted, while AZD0780 remains in Phase I. Novel approaches aim to convert injectable ASOs into oral therapeutics, including AZD6615 (derived from AZD8233) and CIVI 008. These programs leverage innovative nanoparticle delivery systems to overcome gastrointestinal absorption barriers, though currently remain in preclinical stages [254].

#### ANGPTL3 and ANGPTL8 inhibitors

ANGPTL3 and ANGPTL8, secreted glycoproteins sharing structural homologous with angiopoietins, constitute critical modulators of lipid homeostasis [255]. These proteins inhibit lipoprotein lipase (LPL) and endothelial lipase (EL) activities. Pharmacological blockade of ANGPTL3/8 manifests clinically as reductions in plasma triglycerides (TG) and LDL-C.

Evinacumab, a fully human IgG4 monoclonal antibody, demonstrates potent ANGPTL3 neutralization. Intravenous administration (20 mg/kg body weight) of evinacumab resulted in mean reductions of 63% in plasma triglycerides, 28% in LDL-C, and 20% in HDL-C by day 15. In another Phase I study, subcutaneous administration (20 mg/kg, every four weeks) reduced plasma triglycerides, LDL-C, non-HDL-C, and HDL-C by 78%, 35%, 44%, and 8%, respectively, at week 8 [256]. Zodasiran (formerly ARO-ANG3) and vupanorsen, second-generation N-acetylgalactosamine (GalNAc3)-modified ASOs, selectively bind to hepatic *ANGPTL3* mRNA and suppress ANGPTL3 production via potent and sustained mRNA inhibition [257, 258]. Unlike ANGPTL3 inhibitors, the therapeutic potential of ANGPTL8 inhibition has been explored only in preclinical studies [259]. REGN3776 is a fully human monoclonal antibody. A single subcutaneous dose of REGN3776 (10 mg/kg) has been shown to enhance LPL activity and triglyceride clearance, lowering plasma triglycerides. LY3475766 is an anti-ANGPTL3/8 complex monoclonal antibody. Although circulating ANGPTL3/8 complex levels are significantly lower than ANGPTL3 alone, the complex is 100 times more potent at inhibiting LPL activity. A single dose of LY3475766 (10–30 mg IV or 100, 300, or 600 mg SC) led to dose-dependent reductions in plasma triglycerides and LDL-C of up to 70% and 35%, respectively, while increasing HDL-C levels by up to 25%. No serious adverse events or discontinuations due to adverse effects were reported during the study [260].

#### Apolipoprotein C3 (ApoC3)

ApoC3 plays a critical role in lipoprotein metabolism by inhibiting LPL activity and hepatic uptake of triglyceride-rich lipoproteins (TRLs), thereby leading to elevated plasma triglyceride levels. Genetic evidence from loss-of-function mutations demonstrates that ApoC3 deficiency correlates with reduced circulating triglycerides and a lower risk of cardiovascular risk, underscoring its potential for managing hypertriglyceridemia and atherosclerosis. Capitalizing on this genetic insight, antisense oligonucleotide (ASO) therapies have been engineered to specifically degrade ApoC3 mRNA, effectively suppressing protein expression and restoring lipid homeostasis.

The first-generation ASO volanesorsen demonstrated dose-dependent efficacy, achieving 79.6% reduction in ApoC3 levels and 70.9% decrease in triglycerides through enhanced TRL catabolism, while concurrently elevating HDL-C and reducing VLDL-C and ApoB-48. This metabolic remodeling was accompanied by transient LDL-C elevation, attributed to accelerated VLDL-to-LDL particle conversion [261].

To optimize therapeutic precision, the second-generation GalNAc-conjugated ASO AKCEA-APOCIII-LRx was developed, leveraging triantennary N-acetylgalactosamine moieties to achieve 98% hepatic uptake via asialoglycoprotein receptor (ASGPR)-mediated targeting. Clinical trials demonstrated Superior efficacy with 89–92% ApoC3 Suppression and 66–77% triglyceride reduction following either single-dose (120 mg) or chronic dosing regimens (30 mg weekly/60 mg monthly), alongside marked HDL-C elevation and significant reductions in non-HDL-C, VLDL-C, and ApoB [262]. Notably, this targeted delivery system virtually eliminated systemic toxicity, with no reported cases of thrombocytopenia or hepatorenal dysfunction across trials, establishing a safety profile conducive to chronic administration. The evolution from volanesorsen to GalNAc-ASO exemplifies successful translational refinement—enhancing potency through tissue-specific delivery while mitigating off-target risks, positioning ApoC3 inhibition as a transformative strategy for severe dyslipidemias.

#### LXRs

LXRs, comprising the α (NR1H3) and β (NR1H2) isoforms, function as ligand-activated nuclear receptors that act as master regulators of cholesterol homeostasis through integrated metabolic and immunomodulatory mechanisms. These intracellular cholesterol sensors coordinate transcriptional networks mediating RCT while simultaneously suppressing pro-inflammatory pathways, thereby addressing both lipid accumulation and vascular inflammation in atherosclerosis [263]. Upon ligand activation, LXRs induce the expression of cholesterol efflux transporters ABCA1 and ABCG1 alongside apolipoprotein E, creating a coordinated system for mobilizing excess cholesterol from macrophages to hepatic excretion pathways. This RCT potentiation is mechanistically complemented by LXR-mediated transrepression of NF-κB signaling, which downregulates inflammatory mediators including TNFα, IL-1β, and iNOS—a dual-action strategy that disrupts the pathological synergy between lipid deposition and vascular inflammation [264]. Current therapeutic development focuses on overcoming historical limitations through β-isoform selective agonists to avoid hepatic steatosis, nanoparticle-based tissue targeting to enhance vascular specificity, and combination therapies mitigating metabolic side effects. Emerging strategies integrate cryo-EM structural insights into ligand-receptor interactions with advanced delivery platforms, enabling precision engineering of next-generation LXR modulators. These multidisciplinary approaches aim to translate the mechanistic elegance of LXR biology into clinically viable therapies that concurrently optimize cholesterol flux and inflammatory responses in atherosclerotic plaques [265].

#### CETP

CETP plays a pivotal role in lipid metabolism by mediating the exchange of cholesteryl esters (CEs) from HDL to ApoB-containing lipoproteins such as LDL/VLDL in exchange for triglycerides. This lipid redistribution influences plasma lipid composition and has been implicated in the pathogenesis of atherosclerosis [266]. The therapeutic journey of CETP inhibition has undergone significant evolution: early inhibitors like torcetrapib demonstrated potent HDL-C elevation and LDL-C reduction, yet failed due to off-target toxicity including aldosterone-mediated hypertension [267]. Subsequent agents—dalcetrapib and evacetrapib—refined selectivity but failed to reduce major adverse cardiovascular events (MACE) in phase III trials [268, 269], challenging the "HDL hypothesis" and prompting mechanistic re-evaluation. Breakthroughs emerged with anacetrapib, which in the REVEAL trial achieved sustained LDL-C lowering and ApoB reduction, correlating with a MACE risk reduction, suggesting clinical benefit derives from attenuating atherogenic lipoproteins rather than HDL modulation alone [270]. This paradigm shift has catalyzed development of next-generation inhibitors like obicetrapib, featuring optimized pharmacokinetics and increased CETP binding affinity [271]. Concurrently, innovative CETP vaccine strategies are being explored to induce endogenous anti-CETP antibodies [272]. Although still in early stages, this immunotherapeutic approach represents a novel avenue for modulating lipoprotein metabolism [273]. Modern consensus posits that CETP inhibition exerts cardiovascular protection primarily through remodeling ApoB lipoprotein composition (reduced CE content) and enhancing HDL functionality, rather than mere HDL-C quantification, redefining its therapeutic rationale in precision lipidology [274].

#### LCAT

LCAT stands as the sole plasma enzyme responsible for catalyzing cholesterol esterification, driving HDL particle maturation to meet RCT and maintain systemic lipid homeostasis [275]. Therapeutic strategies targeting LCAT encompass three primary modalities: 1) Recombinant human LCAT (rhLCAT) therapies like ACP-501 and MEDI6012 heave demonstrated acute benefits in familial LCAT deficiency (FLD), elevating HDL-C, correcting lipoprotein electrophoretic abnormalities, and improving renal function through intravenous administration. Early-phase trials also suggest potential for reversing HDL dysfunction in acute coronary syndrome, though validation of sustained RCT enhancement and cardiovascular outcomes requires large-scale studies [276, 277]; 2) Gene therapy approaches utilizing adeno-associated virus vectors achieve LCAT overexpression in preclinical models, reducing aortic plaque burden, yet face translational barriers including neutralizing antibody development and limited hepatocyte transduction efficiency. Innovative ex vivo strategies employing LCAT-secreting engineered adipocytes show promise for sustained enzymatic activity but grapple with low long-term cell survival rates [278]; 3) Small-molecule activators such as DS-8190a employ covalent Cys31 modification to boost catalytic activity, while sulfonyl-reactive β-lactams stabilize LCAT-membrane interactions, partially rescuing function in pathogenic LCAT mutants. However, interspecies variability and unresolved allosteric regulation mechanisms constrain clinical application [279, 280].

Despite their notable capacity to elevate HDL-C levels, LCAT-targeted therapies remain controversial with regard to their anti-atherosclerotic efficacy. The heterogeneity of HDL, the complex dynamics between LCAT and lipoproteins, and the narrow focus of current studies on the RCT pathway hinder a comprehensive understanding of their therapeutic mechanisms. Future research should integrate metabolic tracing techniques to elucidate the multifaceted roles of LCAT in lipoprotein remodeling and assess its synergistic potential with CETP inhibitors or statins [281]. Furthermore, investigation into the pathophysiology of acquired LCAT deficiency (e.g., in chronic kidney disease) and the development of novel antibody-based therapies may offer new avenues for expanding clinical applications.

#### Prekallikrein

Plasma prekallikrein (PK), encoded by the *KLKB1* gene, is a circulating protein primarily involved in the contact activation pathway of coagulation and the kallikrein-kinin system. It plays a critical role in inflammation, fibrinolysis, and blood pressure regulation. In recent years, accumulating evidence has highlighted a potential role for PK in lipid metabolism, particularly in cholesterol homeostasis. PK modulates cholesterol metabolism by directly interacting with the LDLR. Studies have shown that PK binding to LDLR promotes lysosomal degradation of the receptor, leading to a reduction in cell surface LDLR levels—especially on hepatocytes—thereby impairing LDL-C clearance and resulting in elevated plasma cholesterol levels [282]. Although the role of PK in cholesterol metabolism has been preliminarily characterized, several challenges remain. First, current findings are largely derived from animal models, and there is a lack of clinical trial data in humans to confirm efficacy and safety. Second, the long-term consequences of PK inhibition—particularly regarding coagulation function—require further investigation. Additionally, the development and clinical translation of PK inhibitors may encounter cost and accessibility constraints, necessitating careful consideration of the balance between therapeutic benefit and economic feasibility.

#### Asialoglycoprotein receptor 1 (ASGR1)

ASGR1 is predominantly expressed in the liver and has emerged as a novel regulator of cholesterol metabolism. Studies indicate that ASGR1 may facilitate the clearance of lLDL, thereby contributing to reduced circulating cholesterol levels. Genetic ablation of *ASGR1* has been shown to enhance the expression of *ABCA1* and *ABCG5/G8* via stabilization of LXRα, promoting cholesterol efflux to HDL and subsequent excretion through bile and feces [283]. Moreover, ASGR1 modulates hepatic cholesterol homeostasis by regulating the expression of genes such as *INSIG1*, thereby suppressing the SREBP pathway and reducing endogenous cholesterol synthesis. Loss of ASGR1 function also inhibits endocytosis and lysosomal degradation of glycoproteins in hepatocytes, leading to a decrease in lysosomal amino acid levels. This nutrient-sensing disruption suppresses mTORC1 signaling while activating AMPK. Activation of AMPK in turn stabilizes LXRα and suppresses SREBP1 activity, further contributing to the downregulation of lipid biosynthesis [284].

Due to its pivotal role in cholesterol metabolism and liver-specific expression, ASGR1 has garnered interest as a promising therapeutic target for hypercholesterolemia and associated cardiovascular disorders [285]. Therapeutic strategies targeting ASGR1 are rapidly evolving and encompass a variety of platforms, including monoclonal antibodies, bispecific antibodies, siRNAs, and ImmunoTAC conjugates. Notably, AMG 529, a human monoclonal antibody developed by Amgen targeting ASGR1, demonstrated favorable tolerability and therapeutic potential in a Phase I clinical trial [286]. However, no updates have been reported regarding its progression to Phase II trials. Additional candidates, such as SZN-043, a bispecific antibody developed by Surrozen, and SBT8230, an ASGR1-TLR8 ImmunoTAC conjugate by Silverback Therapeutics, have also shown encouraging results in preclinical or early clinical development stages [287].

#### Lipoprotein(a) [Lp(a)]

Lp(a), a unique lipoprotein particle structurally analogous to LDL, features an LDL-like core covalently bonded to apolipoprotein(a) through disulfide linkage. Lp(a) has been identified as an independent risk factor for atherosclerotic cardiovascular disease [288]. Despite lacking direct involvement in cholesterol biosynthesis, Lp(a)'s role in lipid transport—particularly its capacity to deliver cholesterol to arterial intima—positions it as a critical therapeutic target in residual cardiovascular risk management..

Several therapeutic strategies targeting Lp(a) are currently under development. Monoclonal antibodies against Lp(a) aim to directly clear circulating Lp(a), though clinical data supporting their efficacy remain limited. Olpasiran, a siRNA agent, lowers Lp(a) levels by inhibiting *LPA* mRNA expression [289]. The OCEAN(a)-DOSE clinical trial demonstrated that a single dose of olpasiran could reduce Lp(a) levels by 70–97%, with effects lasting up to 12 weeks. Similarly, lepodisiran, another siRNA therapeutic, achieved a 94% reduction in Lp(a) levels with a single dose, with the effect sustained for nearly one year. Pelacarsen, an antisense oligonucleotide, suppresses ApoA translation and has been shown to reduce Lp(a) levels by approximately 80% in clinical studies [290]. Muvalaplin, an orally administered small-molecule inhibitor, targets Lp(a) assembly and has demonstrated a 65% reduction in circulating Lp(a) levels in early-phase trials [291]. Furthermore, CRISPR-Cas9 gene-editing strategies targeting the *LPA* gene are under investigation for potentially long-term suppression of Lp(a), although this approach remains in the early stages of preclinical development. These breakthroughs herald a paradigm shift in addressing elevated Lp(a), transitioning it from an intractable risk marker to a modifiable therapeutic target. However, critical questions persist regarding long-term safety of profound Lp(a) suppression (> 80%) and definitive cardiovascular outcome benefits. As more Lp(a)-targeted interventions progress into clinical practice, Lp(a) may evolve from a difficult-to-modify risk factor to a viable and actionable therapeutic target.

#### MicroRNAs (miRNAs)

MiRNAs have emerged as precision regulators of lipid homeostasis, offering novel therapeutic avenues for cardiovascular diseases through their ability to simultaneously coordinate multiple metabolic pathways. For example, anti-miR-33 therapy enhances the expression of *ABCA1* and *ABCG1*, thereby promoting HDL functionality and cholesterol efflux. This approach has demonstrated efficacy in reducing atherosclerotic plaque formation in animal models. Similarly, therapies targeting miR-122, such as *miravirsen*, have been shown in preclinical studies to lower cholesterol levels; however, potential reductions in HDL-C warrant further evaluation. Other miRNAs, including miR-27 and miR-148a, also exhibit therapeutic potential by modulating LDL metabolism and facilitating cholesterol efflux, thus contributing to lipid profile improvement [292, 293]. Nonetheless, miRNA-based therapeutics face several challenges, such as limited delivery specificity, off-target effects, and potential immunogenicity. Current research efforts are focused on the development of nanoparticle-based carriers and chemically modified oligonucleotides to enhance therapeutic efficacy and safety [294]. Despite promising preclinical findings, the clinical application of miRNA-targeted therapies remains contentious. Data from animal models may not fully translate to humans, and long-term safety and efficacy require validation in clinical trials. Additionally, the cell- and tissue-specific variability in miRNA target profiles and functions may influence therapeutic outcomes.

#### Natural products and dietary supplements

Beyond conventional pharmacological therapies, natural products and dietary supplements have emerged as promising adjuncts for hypercholesterolemia management. Emerging studies demonstrate that propionate-enriched diets exert dual immunometabolic effects in murine models, elevating regulatory T-cell populations and IL-10 expression while suppressing jejunal NPC1L1-mediated cholesterol absorption [295, 296]. Notably, probiotic interventions have also exhibited potential: Lactobacillus plantarum H6, isolated from homemade fermented foods in Northeast China, significantly reduced serum cholesterol levels in C57BL/6 mice [297], while Lactobacillus plantarum E680, a novel probiotic from traditional Chinese fermented kimchi, decreased total cholesterol and LDL-C levels in a hypercholesterolemic mouse model [298].

Concurrently, the global dietary supplement market is replete with products that claim to lower cholesterol levels. However, most of these products lack rigorous clinical validation due to regulatory exemptions. Despite this, biochemical studies have confirmed the efficacy of several key agents, including soy protein, green tea, plant sterols, probiotic yogurt, marine-derived omega-3 fatty acids, and red yeast rice. Other products, such as seaweed, berberine, hawthorn, and garlic, may provide limited benefits for specific patient populations. Although these natural products do not lower lipid levels as effectively as statins, most can be safely used in conjunction with lifestyle modifications and pharmacotherapies [299].

Several mechanisms underlie the cholesterol-lowering effects of these natural compounds [300]. Probiotics and soluble fiber bind intestinal cholesterol directly, promoting its excretion via feces [301]. Plant sterols reduce cholesterol absorption by competing with cholesterol for incorporation into micelles [302]. Green tea inhibits cholesterol absorption by suppressing squalene oxidase activity and directly inhibiting micellar cholesterol uptake [303]. Turmeric (curcumin), extracted from the rhizome of turmeric, reduces intestinal cholesterol uptake by inhibiting NPC1L1 expression on intestinal epithelial cells [304]. Soy protein upregulates LDLR, enhancing LDL-C clearance. Berberine, derived from Rhizoma Coptidis, downregulates PCSK9 and increases LDLR expression and LDL uptake [305]. Guggul and hawthorn counteract the downregulation of CYP7A1, enhancing the conversion of cholesterol to bile acids. Omega-3 fatty acids inhibit fatty acid incorporation into triglycerides and enhance triglyceride clearance by increasing LPL activity. Red yeast rice inhibits HMGCR [306]. Garlic inhibits sterol 4a-methyl oxidase, suppressing cholesterol biosynthesis [300].

While these dietary supplements and natural products may not replace pharmacological therapies, they offer complementary benefits for lipid management and overall cardiovascular health. Future research should prioritize standardization of active components, characterization of dose–response relationships, and profiling of potential drug interactions to optimize their therapeutic potential.

## Conclusions and perspectives

Cholesterol and its metabolism are fundamental to cellular homeostasis and systemic health, intricately regulating processes ranging from membrane integrity and steroidogenesis to signal transduction. This review has delineated the molecular machinery governing cholesterol biosynthesis, absorption, conversion, and clearance, emphasizing how dysregulation precipitates pathologies such as atherosclerosis, MAFLD, neurodegenerative disorders, and cancer. Despite revolutionary advances in lipid management through statins, PCSK9 inhibitors, bile acid sequestrants, natural compounds, and emerging siRNA/gene-editing therapies, persistent challenges such as residual cardiovascular risk, statin intolerance, and inadequate efficacy in genetic disorders (notably HoFH) underscore significant unmet clinical needs. Addressing these limitations requires bridging fundamental gaps in our understanding of cholesterol biology, such as spatiotemporal cholesterol trafficking priorities, context-dependent functions of metabolites, and evolutionary paradoxes in pathway conservation. Resolving these mechanistic frontiers will be pivotal for transforming cholesterol biology from a catalogued pathway into a contextually resolved framework for precision medicine. The following key areas represent critical opportunities for future research.

### Spatial regulation and compartmentalization

Despite comprehensive understanding of cholesterol synthesis, efflux, and uptake mechanisms, a critical gap persists in mapping the real-time, spatiotemporal dynamics of cholesterol trafficking across organelles, particularly at membrane contact sites such as ER-mitochondria junctions where cholesterol flux is partitioned between signaling and storage [307]. Resolving this spatial regulation is paramount for explaining tissue-specific pathologies, such as why defective neuronal cholesterol handling drives neurodegeneration while hepatic dysregulation precipitates MAFLD. Closing this knowledge gap will require deploying advanced tools, including super-resolution imaging, fluorescence resonance energy transfer (FRET)-based cholesterol biosensors, and organelle-specific lipidomics, to precisely chart cholesterol’s subcellular itineraries and illuminate how compartmentalization orchestrates its functional diversity [308, 309].

### Inter-tissue cholesterol crosstalk

Complementing the subcellular view, an emerging paradigm centers on deciphering inter-tissue communication networks beyond the canonical liver-intestine axis [310]. Key unresolved questions persist regarding how extrahepatic tissues—including adipose depots, skeletal muscle, and immune cells—orchestrate cholesterol status through endocrine signals such as HDL-borne miRNAs or enzymatically derived oxysterols (e.g., 25-OHC), and how these mediators integrate systemic homeostasis. Elucidating these pathways is critical for explaining organ-specific disparities in therapeutic responses, exemplified by statin-induced myopathy in muscle or neurocognitive effects in the CNS despite systemic LDL-C reduction [7]. To unravel this complex crosstalk, future studies must employ tissue-specific knockout models targeting cholesterol sensors (LXRs, INSIGs) and transport machinery (ABCA1, SR-BI), revealing how distributed tissues collectively govern whole-body cholesterol equilibrium.

### Context-dependent functions of cholesterol metabolites

Beyond cholesterol itself, oxysterols Such as 25-OHC and 27-OHC are now recognized as potent signaling molecules rather than mere metabolic byproducts. However, their context-dependent functions present a critical knowledge gap. Paradoxically, 27-OHC promotes breast cancer metastasis while suppressing glioblastoma growth [311], and 24S-hydroxycholesterol (24S-OHC)—essential for brain cholesterol elimination—simultaneously exacerbates amyloid-β production in Alzheimer’s disease [312]. These divergent roles, dictated by tissue microenvironment and receptor expression, complicate therapeutic targeting of oxysterol-generating enzymes like CYP46A1 or CH25H, as systemic inhibition may yield opposing effects across pathologies. To resolve this, future work must develop tissue-selective enzyme modulators and comprehensively map context-specific receptor interactions (e.g., EBI2 in immunity, RORγt in cancer) to harness oxysterols’ therapeutic potential without unintended consequences.

### Cholesterol-immunometabolism nexus

A closely related and critical underexplored frontier lies at the intersection of cholesterol biology and immunometabolism, where key questions persist about how cholesterol reprograms immune function beyond its established role in lipid raft assembly [313]. This gap is exemplified by macrophage responses to cholesterol crystals—which trigger NLRP3 inflammasome activation and drive atherosclerosis—and T-cell cholesterol enrichment that amplifies TCR signaling to promote autoimmunity. Resolving these mechanisms explains perplexing clinical observations, such as the anti-inflammatory effects of PCSK9 inhibitors that occur independently of LDL-C reduction. To decode this crosstalk, future research must define precise metabolic checkpoints where cholesterol interfaces with immunologic pathways, including STING-mediated inflammation and mTOR-dependent cell activation, thereby revealing new targets for immunomodulatory therapies.

### Evolution perspective on cholesterol homeostasis

Finally, a profound conceptual opportunity arises from evolutionary paradoxes in cholesterol regulation, particularly why humans retain two mechanistically distinct biosynthesis pathways (Bloch vs. K-R) despite their metabolic redundancy [314]. This gap extends to whether these pathways enable tissue-specific stress adaptations—such as K-R’s dominance in skin under UV exposure or hypoxia—and how evolutionary pressures forged species-specific regulatory architectures, exemplified by human *CYP7A1*’s lack of functional LXR response elements unlike rodent orthologs. Resolving these questions could illuminate why cholesterol-linked pathologies like gallstones and Alzheimer’s exhibit human predominance, potentially reflecting trade-offs in our metabolic trajectory. Pursuing this requires comparative genomics of cholesterol pathways across vertebrates and invertebrates, coupled with experimental validation in human organoids, to decode how evolutionary innovations and compromises sculpted modern disease vulnerabilities.

Moving forward, the next era of lipidology must therefore synergize mechanistic insights with clinical innovation, prioritizing precision, durability, and safety beyond broad cholesterol suppression. By advancing contextually optimized multi-target regimens, we may achieve equitable management of cholesterol-related pathologies.

## Data Availability

Not applicable.
